# Stable Translocation Intermediates Jam Global Protein Export in *Plasmodium falciparum* Parasites and Link the PTEX Component EXP2 with Translocation Activity

**DOI:** 10.1371/journal.ppat.1005618

**Published:** 2016-05-11

**Authors:** Paolo Mesén-Ramírez, Ferdinand Reinsch, Alexandra Blancke Soares, Bärbel Bergmann, Ann-Katrin Ullrich, Stefan Tenzer, Tobias Spielmann

**Affiliations:** 1 Bernhard Nocht Institute for Tropical Medicine, Parasitology section, Hamburg, Germany; 2 Institute for Immunology, University Medical Center of the Johannes Gutenberg University Mainz, Mainz, Germany; University of Geneva, SWITZERLAND

## Abstract

Protein export is central for the survival and virulence of intracellular *P*. *falciparum* blood stage parasites. To reach the host cell, exported proteins cross the parasite plasma membrane (PPM) and the parasite-enclosing parasitophorous vacuole membrane (PVM), a process that requires unfolding, suggestive of protein translocation. Components of a proposed translocon at the PVM termed PTEX are essential in this phase of export but translocation activity has not been shown for the complex and questions have been raised about its proposed membrane pore component EXP2 for which no functional data is available in *P*. *falciparum*. It is also unclear how PTEX mediates trafficking of both, soluble as well as transmembrane proteins. Taking advantage of conditionally foldable domains, we here dissected the translocation events in the parasite periphery, showing that two successive translocation steps are needed for the export of transmembrane proteins, one at the PPM and one at the PVM. Our data provide evidence that, depending on the length of the C-terminus of the exported substrate, these steps occur by transient interaction of the PPM and PVM translocon, similar to the situation for protein transport across the mitochondrial membranes. Remarkably, we obtained constructs of exported proteins that remained arrested in the process of being translocated across the PVM. This clogged the translocation pore, prevented the export of all types of exported proteins and, as a result, inhibited parasite growth. The substrates stuck in translocation were found in a complex with the proposed PTEX membrane pore component EXP2, suggesting a role of this protein in translocation. These data for the first time provide evidence for EXP2 to be part of a translocating entity, suggesting that PTEX has translocation activity and provide a mechanistic framework for the transport of soluble as well as transmembrane proteins from the parasite boundary into the host cell.

## Author Summary

*P*. *falciparum* parasites, the deadliest agent of human malaria, develop within erythrocytes where they are surrounded by a parasitophorous vacuolar membrane (PVM). To ensure intracellular survival, the parasite exports a large repertoire of proteins into the host cell. Exported proteins require unfolding for trafficking across the membrane boundaries separating the parasite from the erythrocyte, typical for transport by protein translocating membrane channels. Here, we dissected the sequence of translocation events at the parasite boundary using substrates that can be conditionally arrested at translocation steps. We for the first time obtained exported proteins arrested in the process of being translocated across the PVM. This jammed the translocons for all other types of exported proteins and inhibited parasite growth. The constructs stuck in translocation were in a complex with EXP2, a component of a complex known to be essential for protein export that is termed PTEX. Our work links the need for unfolding and the function of this complex in export, giving experimental evidence that PTEX indeed is a translocon. Conditionally unfoldable domains have been instrumental in unravelling transport processes across membranes and here resolve the transport steps the different kinds of exported proteins require to reach the *P*. *falciparum*-infected host cell.

## Introduction

Five species of *Plasmodium* parasites cause human malaria. Of these *P*. *falciparum* is responsible for the majority of the over 500’000 annually recorded malaria deaths [1]. The pathology of malaria is caused by the continuous propagation of the parasite within red blood cells (RBCs). In this phase *P*. *falciparum* parasites modify extensively the host RBC by exporting hundreds of different proteins into the infected cell. These modifications include host cell surface changes that cause the sequestration of infected RBCs (iRBCs) in the vasculature, a phenomenon considered to be a major contributor to parasite virulence [2]. Other changes are required for nutrient acquisition, to adjust RBC rigidity and to facilitate protein trafficking in the host cell [3]. Protein export is therefore central for blood stage development and malaria pathology.

Two general types of exported proteins have been described in malaria parasites. The first group contains a five amino acid motif termed Plasmodium export element (PEXEL) or host targeting signal (HT) [4–6]. The second group, termed PEXEL negative exported proteins (PNEPs), is defined by the absence of a PEXEL/HT signal [7,8]. Both groups comprise soluble and transmembrane (TM) proteins. Despite the distinction into PNEPs and PEXEL proteins, both types of proteins appear to share a similar export domain [9] and at least at one point during their export, the same trafficking factors are involved [10,11].

Many aspects of the pathways exported proteins use to reach the host RBC are still unclear. The parasite replicates in a parasitophorous vacuole (PV) formed by a PV membrane (PVM) [12]. Exported proteins therefore have to cross two membranes, the parasite plasma membrane (PPM) and the PVM. A previously postulated protein translocation machine termed ‘Plasmodium translocon of exported proteins’ (PTEX) [13], is involved in this export step [10,11]. Of the 5 known PTEX components, heat shock protein 101 (HSP101), and a parasite-specific protein termed PTEX150 are essential for protein export [10,11]. Much less clear is the role of the suspected PTEX membrane pore component EXP2 for which no functional data are available in *P*. *falciparum*. Recent work in the apicomplexan *Toxoplasma gondii* showed that PfEXP2 was able to functionally replace a protein implicated in the solute pore activity at the PVM of this parasite [14]. This raised the possibility that PfEXP2 may have an additional or differing role than in protein export, which would also explain the finding that EXP2, but not HSP101, is expressed in liver stage parasites [15,16].

Although some of its components are clearly essential for protein export, PTEX is still a translocon in concept, as translocation activity has so far not been shown and a function upstream of membrane translocation would also satisfy the findings so far [3,17]. It is also puzzling that it promotes trafficking of both TM and soluble proteins. PTEX is situated on the luminal face of the PVM [13]. While soluble proteins directly reach PTEX after release by transport vesicles into the PV, TM proteins embedded in the transport vesicle membrane will end up integral to the parasite plasma membrane (PPM) [18]. There is evidence that these proteins are then extracted out of the PPM in an unfolding dependent step [9], but the trafficking events at the PPM, PV and PVM remain obscure (reviewed in [17]).

Substrates fused to conditionally foldable domains have been used to study translocation processes in various organelles and systems, for instance in mitochondria [19,20]. A widely used tool for such studies is mouse dihydrofolate reductase (mDHFR), a protein that can be stabilized in its folding upon addition of a small ligand such as WR [19]. If mDHFR is fused to a translocation substrate, addition of the ligand will render the substrate translocation incompetent. The resulting defect in transport is indicative of membrane translocation and excludes vesicular trafficking for the transport step analyzed. This system has previously been used in malaria parasites to show that soluble truncated PEXEL reporters [21], TM PNEPs [9] and soluble PNEPs [7], require unfolding to reach the host cell. However, while clearly indicative of translocation, these experiments did not link this activity with the proposed translocon PTEX.

Here we resolve the translocation steps in the parasite periphery, demonstrating that two unfolding events are required for TM proteins to reach the host cell. Further we show that all known types of exported proteins converge at the second translocation step at the PVM and that inducibly jamming this pore arrests general protein export and parasite development. Crucially, we for the first time obtained stable translocation intermediates and use this to provide evidence that links EXP2 with the translocating complex, suggesting that PTEX has translocation activity.

## Results

### Dissection of unfolding events of exported TM proteins at the parasite periphery indicates a two-step process

The previously used exported TM protein REX2 fused with mDHFR accumulated at the PPM after addition of WR, leading to an arrest at the first step when exported TM proteins leave the parasite cell [9]. In agreement with previous data for a soluble mDHFR fused PEXEL reporter [21], this arrest was not reversible (S1A Fig). This precluded the use of this system to study the subsequent transport steps by simply removing WR. We therefore replaced mDHFR with the bovine pancreatic trypsin inhibitor (BPTI), a protein that forms a translocation incompetent folded structure in oxidising but not reducing environments based on 3 disulfide bonds [20]. The rational was that BPTI would only form a folded structure once the construct reached the PV, which is thought to be an oxidising environment [22,23]. In contrast, extraction out of the PPM, when BPTI still faces the reducing cytoplasmic side of the PPM, would not be affected (see S1B Fig for schematic).

**Fig 1:**
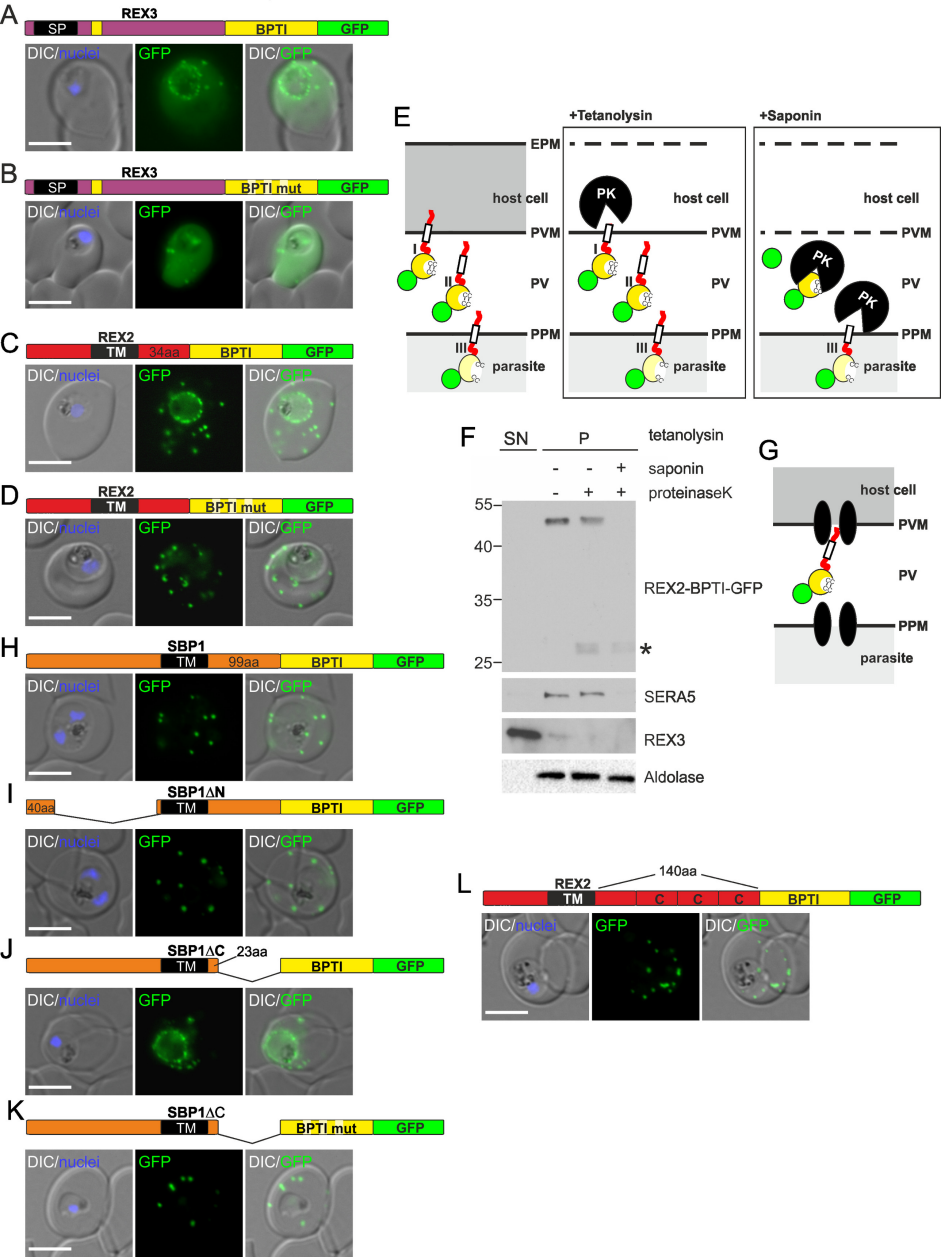
Fusion with the redox sensitive BPTI reveals a second translocation step for TM proteins. (**A-D** and **F-L**) Representative images of live *P*. *falciparum* parasites expressing the constructs shown schematically above each panel. Hydrophobic regions (SP, signal peptide; TM, transmembrane domain) are in black, the PEXEL motif in yellow. Numbers refer to amino acids (aa). Red boxes labelled C, additional REX2 C-termini. Interrupted yellow box, mutated BPTI (BPTImut). DIC, differential interference contrast. Size bars: 5 μm. (**E**) Schematic for the protease K (PK) protection assay. Left, intact infected RBC with 3 possibilities (I, II, III) for the location of the fusion construct: I, protein is integral to PVM; II, protein is freely accessible in the PV; III, protein is integral to PPM. Middle, after permeabilisation of the erythrocyte plasma membrane (EPM) with tetanolysin the N-terminus of the construct will be digested if it is in the PVM (I), but remains intact in situation II and III. Right, after permeabilisation of the PVM with saponin, the constructs will be digested if it is in the PVM (I) or the PV (II) but if in the PPM (III), an N-terminally truncated fragment will be generated. Red, exported protein; white box, TM; yellow, BPTI with double cysteine bonds; green, GFP. (**F**) Western analysis of a protease protection assay according to (**E**). Digestion is visible after saponin treatment only. As no protected fragment is detectable, the protein is freely accessible in the PV (situation II). The faint bands (asterisk) represent protein degraded down to GFP. REX2-BPTI-GFP was detected using anti-GFP antibodies. Control for PVM integrity was SERA5 (PV resident), for the PPM aldolase (resident in parasite cytoplasm). Release of REX3 (resident of host cell cytosol) demonstrated efficient permeabilisation of the EPM. The marker is indicated in kDa. (**G**) Schematic of the location of REX2 based on the protease protection assay shown in (**F**). Translocation machines are indicated as two black ellipses. Other features are as in (**E**).

To first test whether the PV indeed is an oxidative environment where BPTI can fold into a translocation incompetent state and to assess whether PVM translocation was sensitive to this domain, we fused BPTI to REX3, a soluble exported protein that is directly delivered from the secretory pathway into the PV. Export of REX3 was indeed sensitive to BPTI, as evident by a clear, although only partial, accumulation of the construct in the parasite periphery (Fig 1A). In contrast, a REX3 control construct fused with a mutated BPTI [unable to form the stabilising disulfide bridges [24]], did not show an accumulation in the parasite periphery but was fully exported (Fig 1B), excluding oxidation unrelated trafficking defects. This suggested that the system is suitable to obtain translocation incompetent reporters in the PV and we next fused BPTI to the TM PNEP REX2. The resulting construct (REX2-BPTI-GFP) showed a strong accumulation in the parasite periphery (Fig 1C), suggestive of a translocation-dependent step of TM proteins after passing the PPM. This block was not absolute, as additional fluorescence was detected in the Maurer's clefts (Fig 1C). A control construct with a mutated BPTI was fully exported to the Maurer's clefts (Fig 1D), again excluding oxidation unrelated trafficking defects. Protease protection assays (see Fig 1E for schematic) with REX2-BPTI-GFP indicated that the REX2-BPTI-GFP molecules were in the PV in their entireness (Fig 1F) and hence had completed the extraction out of the PPM. These results are consistent with an oxidation state-dependent arrest in export in the PV due to fusion with BPTI (Fig 1G) and indicated that after PPM extraction exported TM proteins undergo a second unfolding-dependent translocation at the PVM.

### The redox sensitive folding domain is only effective if close to the TM

Unexpectedly, when we analysed two further TM PNEPs fused to BPTI (SBP1-BPTI-GFP and MAHRP1-BPTI-GFP), these constructs showed no accumulation in the parasite periphery but were efficiently exported (Fig 1H and S1C Fig). The most noticeable difference between these PNEPs and REX2 is their larger size. We reasoned that protein length might influence whether BPTI can fold in an intermediate step in the PV. To test this idea, we shortened the N- or C-terminus of SBP1 in SBP1-BPTI-GFP by inserting deletions previously reported not to affect export of this protein [25]. The protein with the shortened N-terminus (SBP1ΔN-BPTI-GFP) was not blocked in export (Fig 1I). In contrast, deletion of most of the C-terminus (SBP1ΔCBPTI-GFP) resulted in a strong block in the parasite periphery with some left over export to the Maurer's clefts (Fig 1J), comparable to the result with REX2-BPTI-GFP. This was not due to a general export defect introduced by the C-terminal deletion but due to folding of BPTI, as a version of SBP1ΔC-BPTI-GFP with the mutated BPTI was exported (Fig 1K).

These results suggested that the length of the C-terminus, specifically the region between the TM and the blocking domain, decides whether BPTI has the chance to fold in the PV and the protein gets blocked in further export. To confirm this and exclude an SBP1-specific effect, we extended the C-terminus in REX2-BPTI-GFP by inserting 3 consecutive REX2 C-termini (REX2+3C-BPTI-GFP). This turned REX2 into a protein unaffected by BPTI as this construct was fully exported (Fig 1L). This lends support for a scenario where translocation substrates with a large distance between the TM and the blocking domain already engage the PVM translocon while still being extracted out of the PPM which would prevent release into the PV and oxidation-state dependent folding of BPTI. In contrast, such a direct ‘hand over’ may not be possible for proteins with a short C-terminus where BPTI would already become exposed to the oxidising milieu of the PV to form the folding stabilising disulfide bonds before the protein can get access to the PVM translocon (see model S1D and S1E Fig).

### Certain mDHFR fusions jam the translocon for other exported proteins, leading to a WR-dependent co-block

**Fig 2:**
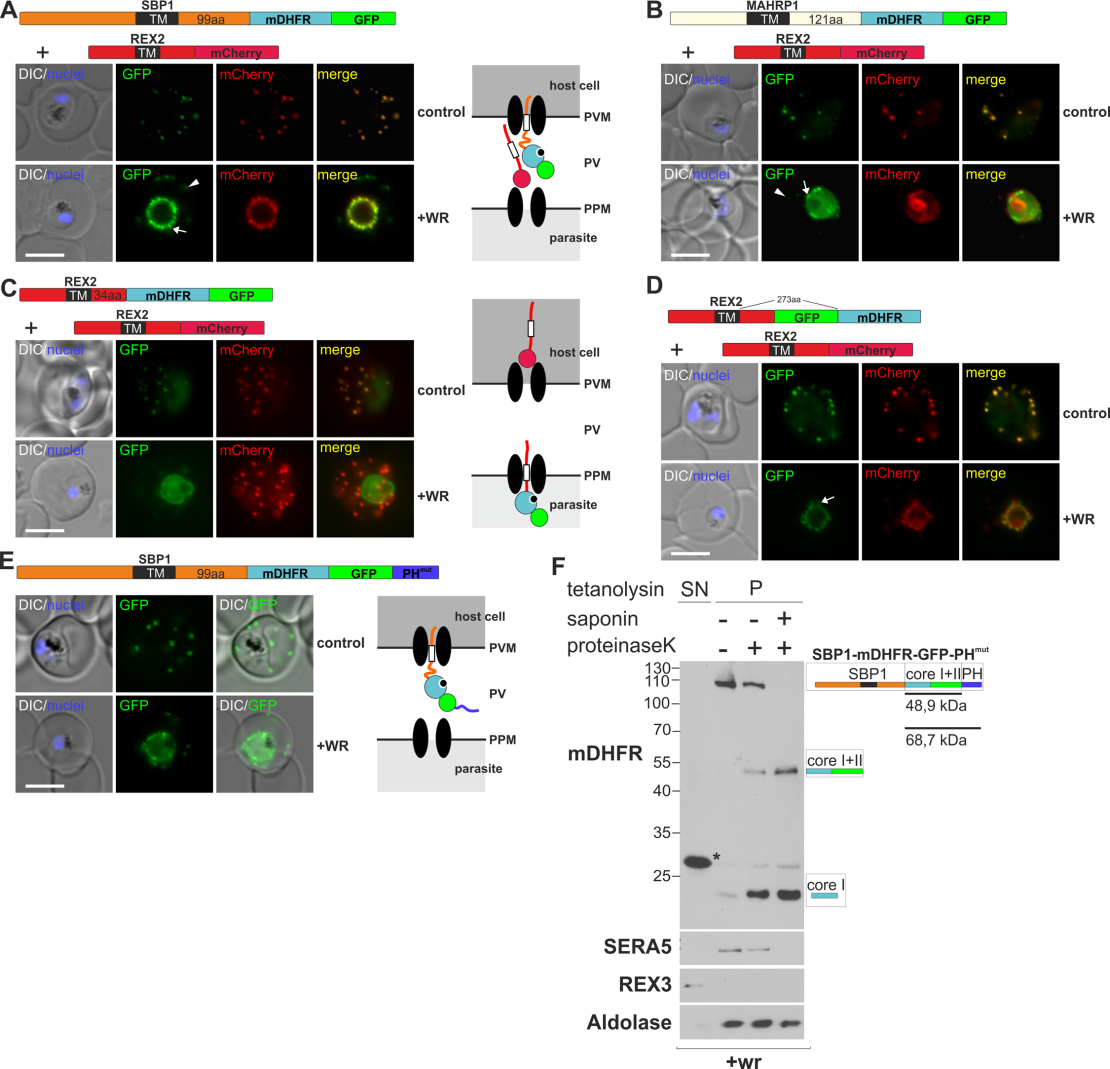
mDHFR fusions can clog the PVM translocon and co-block the export of other proteins. (**A-E**) Representative images of live *P*. *falciparum* parasites grown in the presence (+WR) or absence of WR (control) and expressing the constructs shown schematically above each panel (features as in Fig 1, numbers refer to the length of the amino acids sequence between TM and blocking domain). DIC, differential interference contrast. Arrowheads indicate faint signals at the Maurer’s clefts; arrows show mobile protrusions (note that they do not overlap in the red and the green signal due to movement between capture of the images). Size bars: 5 μm. Schematics to the right show the location of the fusion protein containing the folded WR-bound mDHFR domain (light blue circle with smaller black circle in binding pocket) and the co-expressed REX2 (red line) fused to mCherry (red circle); white box, TM; green circle, GFP; blue line, protease sensitive mutated PH domain. (**F**) Western analysis of a protease protection assay according to the schematic in Fig 1E with WR treated parasites expressing SBP1-mDHFR-GFP-PH^mut^. The construct (detected with α-mDHFR antibodies) is in the PV, as only full length protein or protease resistant cores (after saponin treatment) but no protected fragment indicative of an intact C-terminus is detectable. Calculated molecular weights are, 105 kDa for the full length construct, 48.9 kDa for core I+II and 68.7 kDa for I+II+PH. The asterisk indicates a band likely representing RBC derived hDHFR. Controls are as in Fig 1F. The marker is indicated in kDa.

Prompted by the differences seen with different PNEP-BPTI constructs, we fused SBP1 and MAHRP1 with mDHFR to confirm that they at all require unfolding to pass from the parasite into the host cell. Analogous to REX2-mDHFR-GFP [9], these constructs were efficiently exported to the Maurer's clefts and conditionally blocked in the parasite periphery in the presence of the ligand WR that prevents unfolding of the appended mDHFR domain (Fig 2A and 2B). This indicated translocation as the mode of export for these TM PNEPs, similar to REX2. However, compared to REX2-mDHFR-GFP ([9] and Fig 2C), there were three notable differences in these constructs after arresting export with WR: firstly, the arrest phenotype was leaky in many cells, i.e. besides the prominent peripheral stain, there was also a detectable signal at the Maurer's clefts (arrowheads Fig 2A and 2B). Secondly, the fluorescence pattern in the parasite periphery was unusual as it included small, mobile, worm-like protrusions reaching into the host cell (arrows Fig 2A and 2B). Thirdly and most remarkably, the internal control (co-expressed REX2mCherry) also showed a WR-dependent block in export even though it lacked an mDHFR domain (Fig 2A and 2B, compare to Fig 2C). REX2 fusion with an inverted order of GFP and mDHFR (REX2-GFP-mDHFR, generated in a failed attempt to obtain a reversible mDHFR-based block), showed a similar phenotype to SBP1 and MAHRP1 and differed from REX2-mDHFR-GFP (Fig 2D). Hence, the difference observed was not specific for the exported protein used.

These data indicated that in contrast to REX2-mDHFR-GFP, the export-blocked version of these constructs remained arrested in a translocon that also trafficks REX2mCherry and that arresting these constructs in the process of translocation thereby prevented the passage of REX2mCherry. This effect was clearly caused by the mDHFR fusion protein, as in a subpopulation of cells not expressing the mDHFR construct, REX2mCherry was correctly trafficked to the Maurer's clefts in the presence of WR (S2A Fig). In contrast, REX2mCherry was always arrested in parasites harbouring the GFP-tagged mDHFR fusion (S2A Fig). We termed this effect a 'co-block' as it was caused by a protein fused with mDHFR that was arrested as a stable intermediate in the translocon and prevented passage of the fully translocation competent mCherry control.

The export-arrested form of REX2-mDHFR-GFP, which does not cause a co-block, was previously found to be located at the PPM [9]. We investigated the site of arrest for the co-blocking SBP1-mDHFR-GFP. Initial protease protection experiments indicated that the protein is neither in the PPM nor PVM but in the PV. This was based on the finding that after permeabilisation of the PVM with saponin, addition of protease K digested SBP1-mDHFR-GFP down to its protease resistant core (mDHFR-GFP) which is only possible if this protein is freely accessible in the PV (S2B Fig). However, due to the proportionally small size difference of this core compared to the protected fragment (if the protein were inserted up to the blocking domain into the PPM), we increased the size of the potential protected fragment by appending a protease sensitive domain [a mutated PH domain [26]] to the C-terminus of the construct (Fig 2E). Similar to SBP1-mDHFR-GFP this construct (SBP1-mDHFR-GFP-PH^mut^) was fully exported and conditionally arrested in the parasite periphery after addition of WR (Fig 2E). Protease protection assays showed that in the blocked state the C-terminal PH part was also fully protease accessible in the PV, indicating that the SBP1-mDHFR-GFP-PH^mut^ molecules had entirely passed the PPM (Fig 2F). Hence, the site of block differed from that of REX2-mDHFR-GFP.

The co-blocked REX2-mCherry control was also found in the PV (S2B Fig). The presence of the co-blocked molecules in the PV indicated that the site of arrest of the co-blocking construct is the PVM and that PPM extraction is not prevented by clogging the PVM translocons. This further supports a two-step model of translocation for TM proteins.

### All types of exported proteins are affected by the co-block, indicating that they pass through a single type of pore

**Fig 3:**
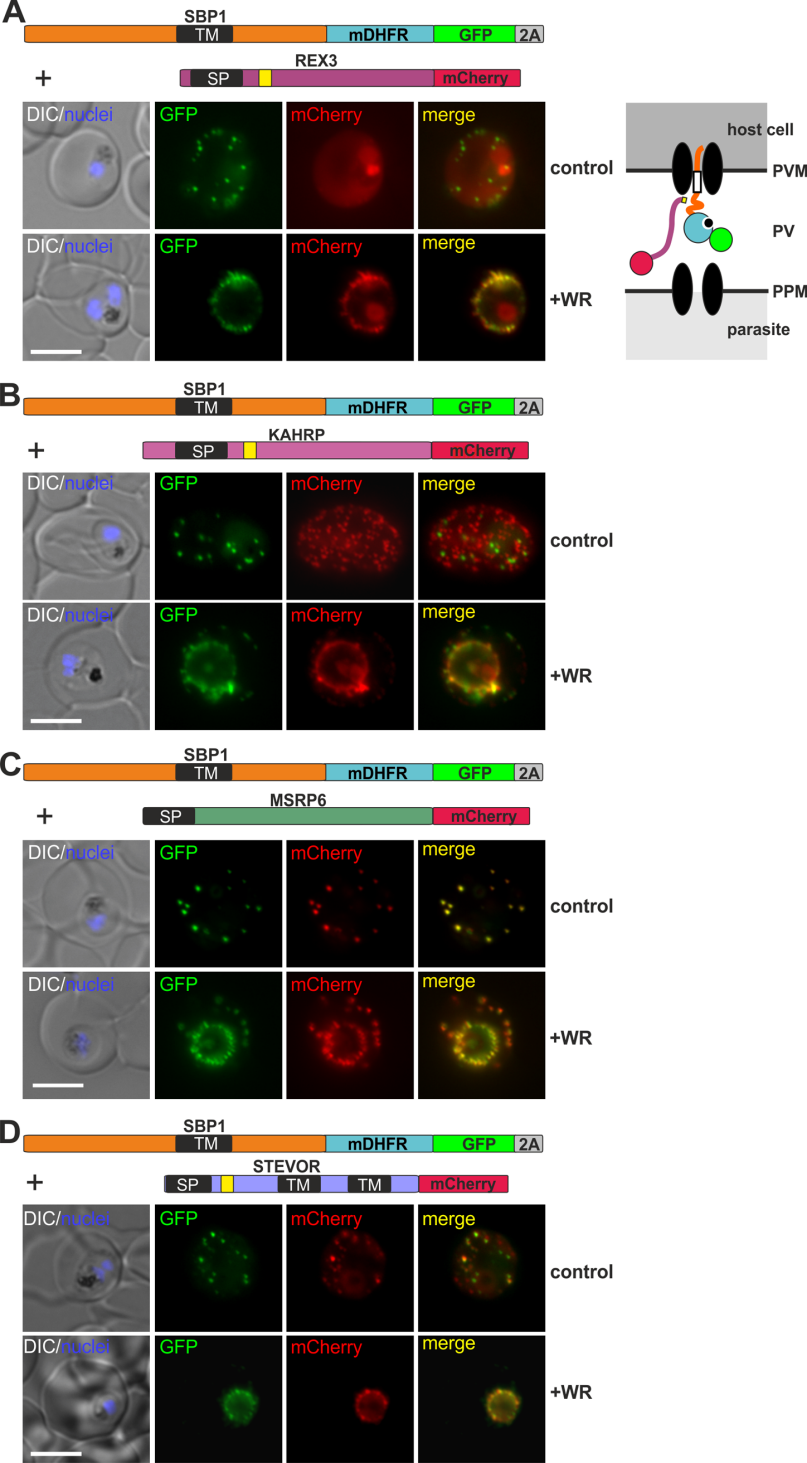
Different types of proteins pass through the same translocon. (**A-D**) Representative images of live *P*. *falciparum* parasites grown in the presence (+WR) or absence of WR (control), expressing the constructs shown schematically above each panel. The skip peptide is indicated by a grey box labelled 2A, other features are as in Fig 1. The two proteins expressed from the same open reading frame are shown skipped (see also S3 Fig). DIC, differential interference contrast. Size bars: 5 μm. A schematic of the co-block is shown for A. The yellow box indicates the mature PEXEL N-terminus, other features are as in Fig 2.

Next we tested whether other kinds of exported proteins besides REX2mCherry were co-blocked by PNEP mDHFR-fusions arrested in translocation. To this end we generated doubly transfected parasites as well as parasites expressing two individual proteins from the same open reading frame using a skip peptide [[27,28], see S3 Fig for demonstration of suitability of this approach] to co-express SBP1-mDHFR-GFP with mCherry tagged members of each of the different known groups of exported proteins. The co-expressed proteins included the soluble PEXEL proteins REX3 and KAHRP, the soluble PNEP MSRP6, and the TM PEXEL protein STEVOR. In each case the co-expressed mCherry fusion protein was hindered in export if SBP1-mDHFR-GFP trafficking was arrested with WR in the translocon (Fig 3A–3D). Similarly, REX2-GFP-mDHFR (the domain order that in contrast to REX2-mDHFR-GFP led to a co-block of REX2mCherry) caused a co-block of the PEXEL protein KAHRP (S4A Fig). These data were also confirmed with endogenous exported proteins detected by IFA using specific antisera: a WR-dependent co-block of the late expressed MSRP6 and KAHRP was seen in SBP1-mDHFR-GFP expressing cells (S4B Fig). In contrast, proteins expressed earlier in the cycle (before SBP1-mDHFR-GFP under the *crt* promoter was expressed), were unaffected (S4C Fig). Taken together, these data show that TM PNEPs arrested during translocation hinder the passage of all known types of exported proteins, indicating that a single kind of protein conducting pore is used by all exported proteins to cross the PVM.

### PEXEL TM proteins require unfolding for export and PEXEL proteins can also cause a co-block

**Fig 4:**
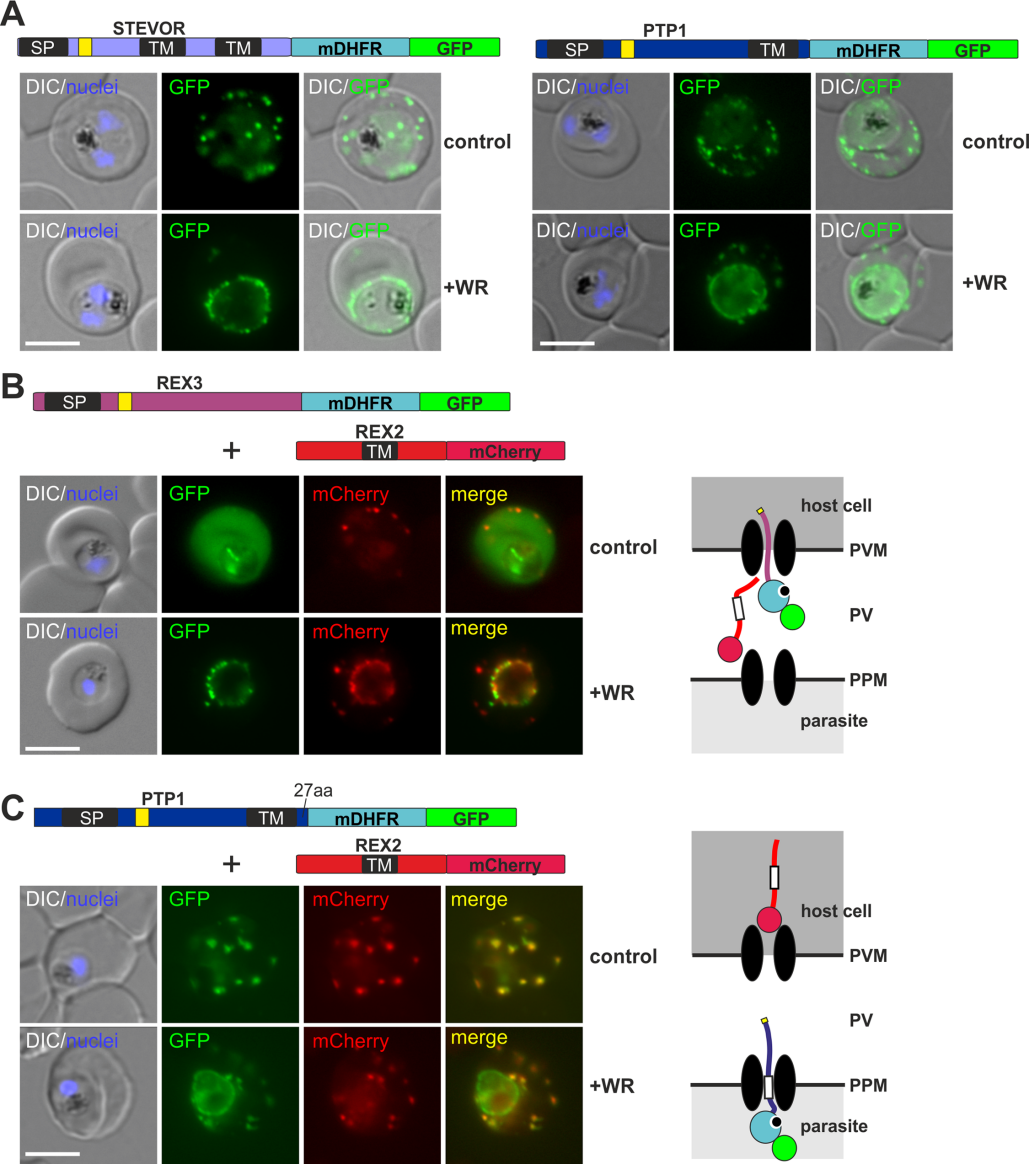
PEXEL TM proteins require translocation for export and some PEXEL proteins can also jam the translocon. (**A-C**) Representative images of live *P*. *falciparum* parasites expressing the constructs shown schematically above each panel (features as in Fig 1, numbers refer to the length of the amino acids sequence between TM and blocking domain). DIC, differential interference contrast. Size bars: 5 μm. A schematic of the co-block (**B**) and failure to co-block (**C**) are shown to the right (features as in Fig 2).

So far it was not tested whether PEXEL TM proteins also require unfolding for export. Our data indirectly indicated that they require translocation, as they were co-blocked by arrested mDHFR fusions, suggesting passage through the same pore. A shared pathway was also indicated by their sensitivity to inactivation of PTEX components [10,11]. Indeed, when PTP1 [a PEXEL protein with a single predicted TM [29]] and STEVOR [two predicted TMs [30]] were expressed as mDHFR-fusions, their export was conditionally arrested when WR was added (Fig 4A). Similar results were obtained with the full length soluble PEXEL protein KAHRP (S5 Fig). Together with the previous data [7,9,21] this indicates that all known types of exported proteins require a membrane translocation step when they pass from the parasite into the host cell.

Next we tested whether it is possible to obtain arrested translocation intermediates of PEXEL proteins that induce a co-block. To this end we generated double transfectants expressing either REX3-mDHFR-GFP or PTP1-mDHFR-GFP together with the TM PNEP REX2mCherry. In the case of REX3-mDHFR-GFP, addition of WR caused a co-block of REX2mCherry (Fig 4B). These results show that other types of proteins than TM PNEPs can induce a co-block. As REX3 is directly released into the PV, the PVM translocon has to be the site where the translocation intermediates are arrested and cause the co-block, consistent with the data obtained with SBP1-mDHFR-GFP (Fig 2).

In contrast to the result with REX3-mDHFR-GFP, no co-block was observed with PTP1-mDHFR-GFP (Fig 4C). It is noteworthy that PTP1-mDHFR-GFP in WR treated parasites did not show worm-like protrusions extending from the PVM, similar to REX2-mDHFR-GFP which is also not co-blocking.

### The capacity to induce a co-block and the sensitivity of export to fusion with BPTI depends on similar properties

**Fig 5:**
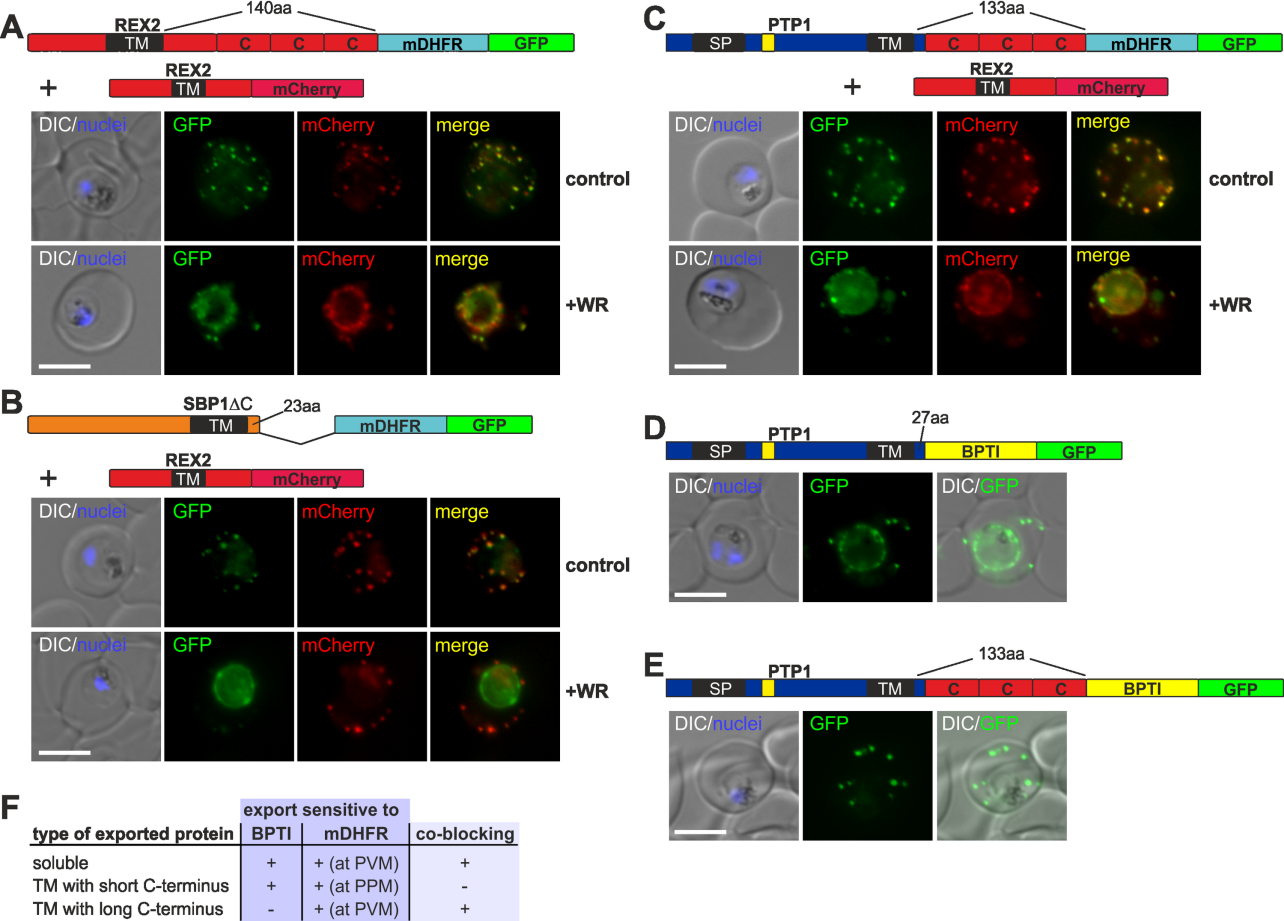
The distance between blocking domain and TM is important for co-blocking and BPTI sensitivity. (**A-E**) Representative images of live *P*. *falciparum* parasites expressing the constructs shown schematically above each panel (features as in Fig 1, numbers refer to the length of the amino acids sequence between TM and blocking domain). (**A-C**) Parasites grown in the presence (+WR) or absence of WR (control). DIC, differential interference contrast. Size bars: 5 μm. (**F**) Table showing the properties of the different types of constructs.

Of the constructs tested so far, those with the capacity to cause a co-block were also insensitive to fusion with BPTI (Figs 1 and 2). Prompted by this correlation, we tested whether the capacity to induce a co-block (as judged by co-expression with REX2mCherry) also depended on the length of region between the TM and the blocking domain. Indeed, extension of this region in REX2 (REX2+3C-mDHFR-GFP) turned this protein into a co-blocker (Fig 5A) whereas shortening this region in SBP1 (SBP1ΔCmDHFR-GFP) changed it into a non-co-blocking protein (Fig 5B). Next we tested whether this was the reason for the failure of PTP1-mDHFR-GFP to induce a co-block. PTP1 has a short C-terminus of 27 amino acids. Extension of the PTP1 C-terminus in this construct (PTP1-mDHFR+3C-GFP) turned this protein into a co-blocker (Fig 5C). Similar to REX2 (Fig 1), export of PTP1 was sensitive to BPTI (Fig 5D), whereas the version with the extended C-terminus (PTP1-BPTI+3C-GFP) again was insensitive (Fig 5E).

These findings support the idea that long C-termini enable engagement with the PVM translocon during PPM extraction and that this is responsible for both, failure of BPTI folding and induction of the co-block (Fig 5F, S1D and S1E Fig). This also further emphasises the similarities in the trafficking modalities of PNEPs and PEXEL proteins, as it affects both groups of proteins alike.

### A pan-export block hampers *in vitro* parasite growth

**Fig 6:**
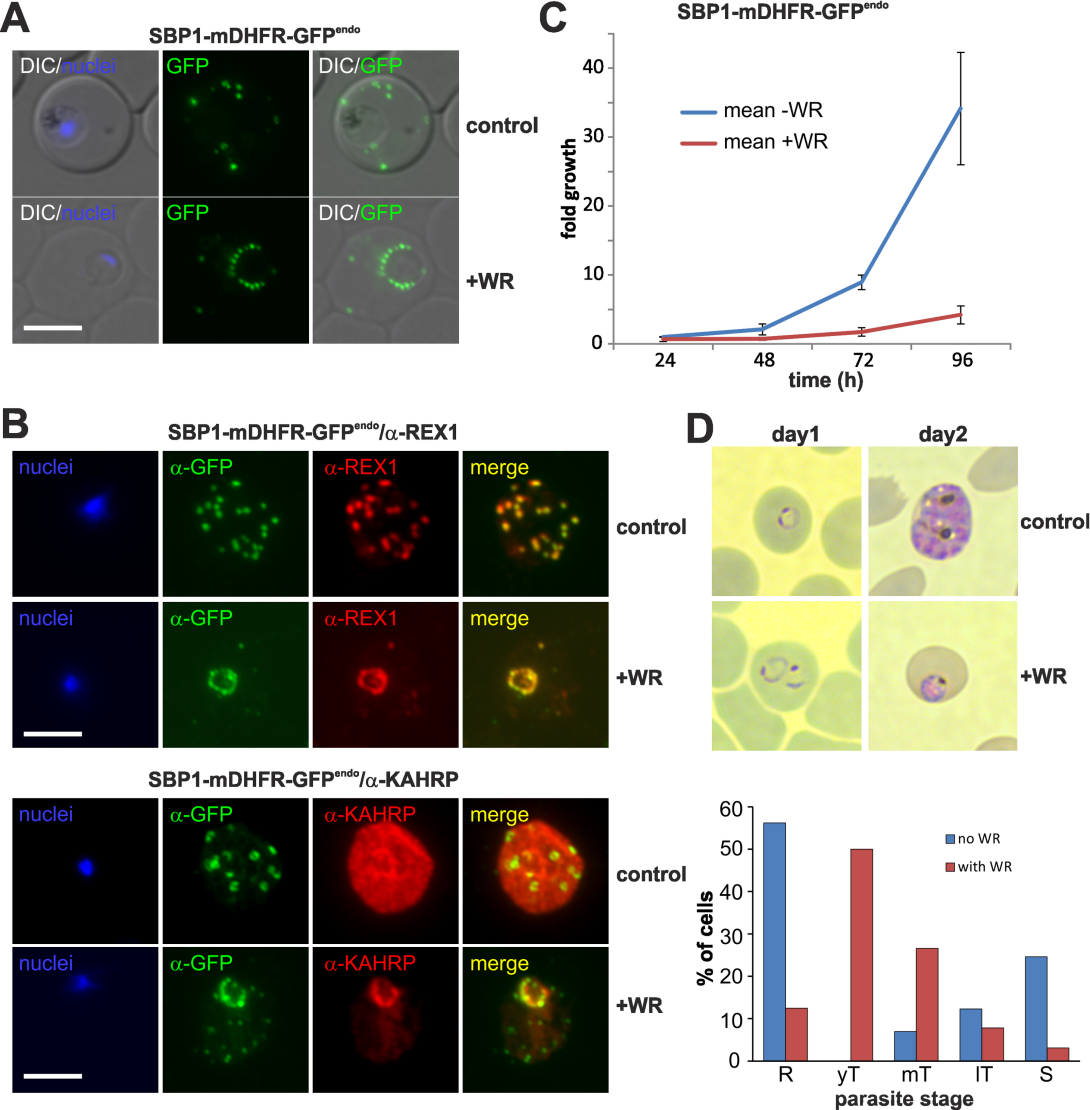
Translocation arrested substrates induce a pan export block that slows parasite development. (**A**) Representative live fluorescence images of the cell line expressing endogenous SBP1 fused to mDHFR-GFP (SBP1-mDHFR-GFP^endo^) grown with (+WR) and without WR (control). DIC, differential interference contrast. Size bar: 5 μm. See also S6 Fig. (**B**) IFA of SBP1-mDHFR-GFP^endo^ parasites using α-REX1 and α-KAHRP in parasites treated with (+WR) and without (control) WR. Size bars: 5 μm. (**C**) Fold growth compared to starting parasitemia of SBP1-mDHFR-GFP^endo^ parasites on (+WR) and off WR (control). Mean of n = 3 independent experiments; error bars represent SD. (**D**) Slowed growth of SBP1-mDHFR-GFP^endo^ parasites in presence (+WR) compared to control (-WR) leads to an accumulation of young trophozoite stages 2 days after addition of WR. Top, Giemsa images (cropped image of larger area shown in S6D Fig); bottom, graph showing stage distribution in cultures after 2 days on WR compared to control (one representative of n = 3 experiments). R, rings; yT, mT, lT: young, mid and late trophozoites, respectively; S, schizonts.

The jammed translocon prevented export of all so far tested exported proteins and provided the opportunity to apply an inducible pan-export block. To assess the effect of generally blocking protein export on parasite growth, we generated an integration parasite line that expresses SBP1 fused to mDHFR-GFP from the endogenous locus (S6A and S6B Fig). We chose SBP1, because it is early expressed [31] and has no essential role for *in vitro* growth [32,33]. The resulting protein, SBP1-mDHFR-GFP^endo^, was correctly trafficked to the Maurer's clefts and arrested after addition of WR (Fig 6A). As this protein was expressed much earlier in the cycle than the mDHFR fusions under episomal *crt* control, a co-block was now also observed for early expressed endogenous proteins, such as REX1, REX2 and MAHRP2, in addition to late expressed proteins like KAHRP (Fig 6B and S6 Fig). Furthermore, the use of an integration cell line ascertained that all cells expressed the co-blocking construct. Growth assays showed that the parasites with the arrested SBP1-mDHFR-GFP^endo^ had a strongly reduced growth rate compared to control (Fig 6C). Giemsa stained smears of export blocked and control cultures revealed a delayed parasite development evident by the accumulation of young trophozoite stage parasites in the export-blocked culture, whereas the controls grew normally (Fig 6D and S6D Fig).

### Substrates stuck in translocation are in a complex with the PTEX component EXP2

**Fig 7:**
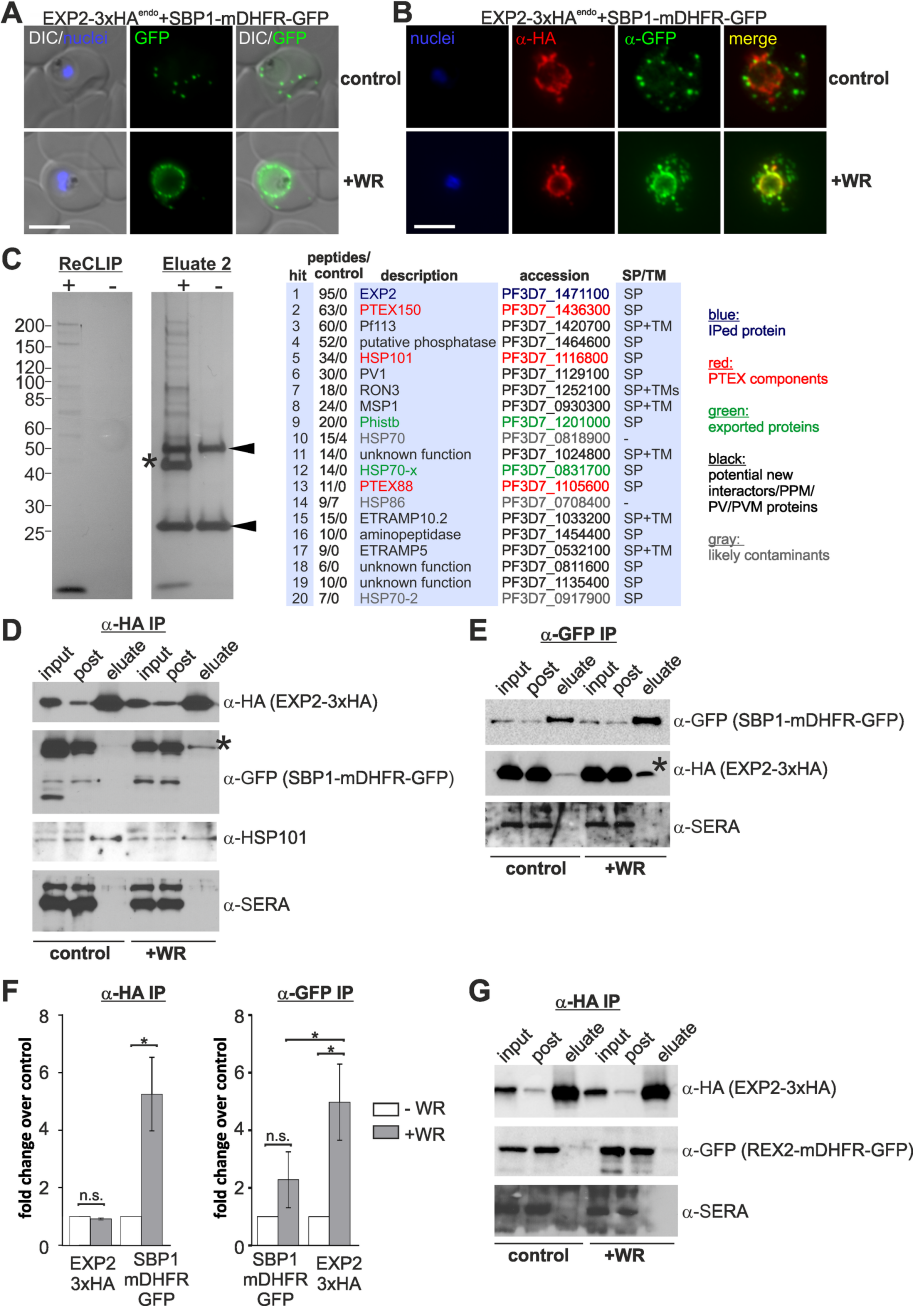
Translocation intermediates are in a complex with EXP2. (**A-B**) Representative live fluorescence (**A**) or IFA (**B**) images of the cell line expressing endogenous EXP2 fused to 3xHA (EXP2-3xHA^endo^) and SBP1-mDHFR-GFP (episomal) grown with (+WR) and without WR (control). DIC, differential interference contrast. Size bars: 5 μm. (**C**) IP of DSP-treated EXP2-3xHA^endo^ parasites (+) using HA binding beads compared to 3D7 parasites (-). Released crosslink eluates (ReCLIP) and NaOH eluates (Eluate2) were separated by SDS-PAGE and silver stained. Asterisk, shows EXP2-3HA; arrowheads show antibody chains. The table shows the 20 top hits after mass spectrometry analysis of a ReCLIP and an Eluate 2. Peptides/control show peptide counts of the indicated protein over 3D7 (ReCLIP and Eluate 2 peptide counts pooled). SP, signal peptide; TM transmembrane domain. (**D**) Western blots of a representative IP experiment using HA binding beads show co-purification of SBP1-mDHFR-GFP (asterisk) in parasites grown with WR but not in untreated controls. Input, total lysate before IP; post, lysate after IP. (**E**) Western blots of a representative IP experiment as in (**D**) but using GFP binding beads to bind SBP-mDHFR-GFP. Asterisk: enrichment of co-purifying EXP2-3xHA in parasites grown with (+WR) compared to control (no WR). (**F**) Quantification of the signal intensity of the IPed EXP2-3xHA and the co-purifying SBP1-mDHFR-GFP (left, asterisk comparing SBP1-mDHFR-GFP in WR+ over its control: p = 0,0288; paired, two-tailed t test; n = 3) or the IPed SBP1-mDHFR-GFP and the co-purifying EXP2-3xHA (right, asterisk comparing EXP2-3xHA in WR+ over its control: p = 0,035; paired, two-tailed t test. Asterisk comparing EXP2-3xHA enrichment with SBP1-mDHFR-GFP enrichment: p = 0,047; unpaired, two-tailed t test; n = 3) in parasites grown with (WR+) or without (control) WR. Error bars show S.D., n.s., not significant. (**G**) Western blots of a representative IP experiment with REX2-mDHFR-GFP expressed in EXP2-3xHA^endo^ parasites. REX2-mDHFR-GFP is not enriched in parasites grown with WR over untreated controls. Input, total lysate before IP; post, lysate after IP.

Translocation activity has so far not been demonstrated for PTEX and the functional role of the proposed pore component EXP2 is unclear [14]. We took advantage of our constructs stuck in translocation to determine whether this arrest involves the proposed translocation pore EXP2 using immunoprecipitations (IP). To this end we generated a cell line expressing 3xHA tagged EXP-2 from the endogenous locus (EXP2-3xHA^endo^) and further transfected it with SBP1-mDHFR-GFP (Fig 7A, S7A and S7B Fig). As expected, treatment with WR led to an arrest of export of SBP1-mDHFR-GFP in the parasite periphery where it co-localised with EXP2-3xHA by IFA (Fig 7B), as previously shown for the PEXEL leader of GBP fused to mDHFR-GFP, a construct that also co-localised with EXP2 when arrested in export [34].

As to our knowledge PTEX was never defined using IP with EXP2-HA, we first wished to confirm that EXP2-3xHA indeed pulls down other PTEX components. IP using anti HA-beads with EXP2-3xHA^endo^ parasites treated with the protein crosslinker DSP followed by mass spectrometry analysis of the eluates revealed the PTEX components PTEX150, HSP101, and PTEX88 as well as several other proteins that may include further PTEX interaction partners (Fig 7C and S3 Table). Hence, EXP2-HA was part of the expected PTEX complex [13]. Next we IPed EXP2-3xHA from parasites that expressed SBP1-mDHFR-GFP in presence of WR (to arrest SBP1-mDHFR-GFP in the translocon) and absence of WR (control). While EXP2-3xHA co-IPed SBP1-mDHFR-GFP when this protein was stuck in translocation, this was not the case in the control (Fig 7D, see Fig 7F for quantification of enrichment). SERA5, a soluble molecule of the PV, to control for non-specific interactions, was not co-IPed. No DSP was used for these experiments but similar results were obtained when DSP treated parasites were used (S7C Fig). This indicated that substrates arrested during translocation are in a complex with EXP2.

To confirm these findings we carried out the reciprocal experiment and tested whether the substrate (SBP1-mDHFR-GFP) arrested in the translocon co-IPed the proposed pore component EXP2-3xHA. EXP2-3xHA was indeed enriched after IP of translocon-arrested SBP1-mDHFR-GFP if compared to control parasites without WR (Fig 7E). As SBP1-mDHFR-GFP also showed some enrichment in WR+ over control after the IP (potentially due to greater stability of the folding stabilised molecule), we quantified the band intensities which showed that this was not significant. In contrast the enrichment of the co-IPed EXP2-3xHA was significant (n = 3, Fig 7F). Taken together these results indicate that SBP1-mDHFR-GFP stuck in translocation is found in a complex with EXP2. In order to confirm this with a second co-block inducing protein, we repeated the anti-HA IP with EXP2-3xHA^endo^ parasites expressing REX2-GFP-mDHFR. Again this substrate was detected in the WR+ eluate but not in the control, demonstrating co-purification with EXP2-HA when this substrate was arrested during translocation (S7D Fig). In contrast, EXP2-3xHA^endo^ parasites expressing REX2-mDHFR-GFP (which is not co-blocking and arrests at the PPM extraction step preceding PTEX), did not co-IP this protein in WR-treated parasites compared to control (Fig 7G). Hence, only the second of the two consecutive unfolding dependent events involves EXP2.

A further candidate of the PTEX components that might interact with the arrested substrate is HSP101, the ATPase that may unfold the substrate. To test whether this is the case or not, we generated a cell line expressing 3HA-tagged HSP101 from the endogenous locus (S7E and S7F Fig) and co-transfected a construct expressing SBP1-mDHFR-GFP. Unlike EXP2-3xHA, HSP101-3xHA did not pull down the substrate after anti-HA IP (S7G Fig). Whilst this could indicate a disassociation of HSP101 from PTEX when substrates are arrested in the translocon, some HSP101 was still interacting with EXP2 in the substrate-arrested state as judged by its presence in the EXP2-3xHA IPed fraction (Fig 7D). Hence, whilst some HSP101 still appears to be part of the complex containing the arrested substrate, it does not seem to be in direct contact with the substrate.

## Discussion

Here we for the first time obtained intermediates of exported proteins inducibly and stably arrested during translocation into the host cell. Intriguingly, these translocation intermediates prevented the transport of all known types of exported proteins, demonstrating that the actual translocation is a point of convergence for all exported proteins and a single kind of protein-conducting channel mediates export. Our data further support a two-step translocation process for exported TM proteins which are first extracted out of the PPM and then translocated into the host cell in a second unfolding-dependent process at the PVM.

Blocking translocation of all exported proteins across the PVM made possible to inhibit general protein export and strongly reduced parasite growth. Similarly to the phenotype observed when HSP101 and PTEX150 were knocked down [10,11], we observed slowed parasite development and an accumulation of young trophozoite stage parasites, although the growth arrest was not absolute. Crucially, our IP data now link the proposed PTEX pore component EXP2 with the entity carrying out the translocation step, fitting with the site of block and the proposed function of PTEX [10,11,13]. Our substrate was arrested in the process of being translocated by preventing unfolding of its C-terminal fusion part. The fact that other proteins could then not pass the PVM clearly shows that the arrested substrate remained in the translocon, likely partly inserted into the membrane channel. Hence, our data support the idea that EXP2 is part of the translocation pore and that PTEX has translocation activity, although it cannot fully exclude a different role of EXP2 in the complex, especially as the link between substrate and EXP2 may also be indirect. However, if there is an indirect link, it does not seem to be via HSP101, as we failed to detect an interaction of arrested substrate with this protein. It should also be noted that we cannot formally rule out that EXP2 at the same time also interacts with a complex other than PTEX that in actual fact carries out the translocation in which case PTEX would have a different, translocon-proximal essential function in export. Finally it is noteworthy that in contrast to the co-blocking SBP1-mDHFR-GFP and REX2-GFP-mDHFR no interaction was detected with export blocked REX2-mDHFR-GFP, a construct that is not co-blocking and arrests at the PPM [9], demonstrating the specificity of the IP results.

Recent data highlighted the possibility of an alternative or dual role of EXP2 as part of a solute transporter [14,16] both of which would be congruent with recent findings indicating that EXP2 is important for the growth of *P*. *berghei* in mice [15]. Our data points to a role in protein export for EXP2 but cannot exclude a dual role. In this respect it is noteworthy that our IP data identified further potential interaction partners of EXP2 and it will be interesting to determine whether these constitute further components of PTEX, interaction partners that (together with EXP2) form a second type of pore conducting solutes or are simply proteins abundant in the PV crosslinked to PTEX by chance.

Two findings indicate that fitting with the location for PTEX, the PVM is the site where the co-block inducing translocation intermediates are arrested. Firstly, a soluble PEXEL protein (a type of protein directly released into the PV that requires translocation at the PVM but not the PPM), also induced a co-block. Secondly, exported TM proteins accumulated in the PV when they were co-blocked, indicating that their extraction out of the PPM was not affected. It should be noted that also the co-blocking construct itself will be co-blocked when all translocation sites are clogged and it can be assumed that this population of the construct by far exceeds the population stuck in translocons.

Our data provide mechanistic insights into the translocations of TM proteins at the PPM and the PVM. The importance of the distance between the blocking domain and the TM (henceforth named spacer) for (i) sensitivity to fusion with BPTI and (ii) the co-blocking capacity of a construct is particularly intriguing. mDHFR fusions with a short spacer were arrested in the PPM [9] and were not co-blocking whereas mDHFR fusions with a long spacer were arrested in PTEX at the PVM, causing a co-block. While other scenario can also be envisaged, we favor two, non-mutually exclusive, possibilities why only proteins with a long but not a short spacer reach the PVM translocon and prevent the export of other proteins. As not the total length from the N-terminus to the blocking domain but specifically the distance between blocking domain and TM domain was important, the TM domain must play a role in this effect. The first possibility is that the TM domain is part of the recognition signal that has to emerge far enough out of the PPM extractor to become available for engagement with PTEX, leading to a transient interaction and hand-over of the substrate. This idea is supported by the fact that the type of TM region is one critical determinant for a protein to be exported [9,25,35]. The second possibility is that a short spacer keeps the TM domain in the PPM extractor close to the membrane milieu which could favor lateral release into the membrane if extraction is blocked.

The BPTI results also support the hand-over model, as in proteins with long spacers, BPTI is not exposed to the oxidizing environment of the PV and cannot fold, suggesting that the polypeptide chain is passed from the PPM extractor directly through PTEX (S1D and S1E Fig). Taken together the different properties of mDHFR and BPTI constructs indicate that TM proteins with a short C-terminus have a brief intermediate in the PV, similar to soluble proteins, and that TM proteins with a long C-terminus cause a transient interaction of the PPM and PVM protein conducting machines while being translocated into the host cell. This is similar to the situation in mitochondria where the translocators of the outer and inner membrane transiently interact during substrate transport [36]. However, this does not appear to be mandatory in the parasite, as proteins with a short spacer were not directly handed over. From our experiments it can be assumed that a spacer as short as 99 amino acids (C-terminus of SBP1) but longer than 34 amino acids (C-terminus of REX2) enables the TM of the substrate to emerge sufficiently from the PPM to engage PTEX at the PVM and to induce a direct hand over without intermittent release into the PV. Finally, other models for the observed phenotypes and for the translocations at the PPM and PVM may also be possible and it will be interesting to see how future work conforms with the here favored scenario.

It is interesting that mDHFR fused export substrates only block the passage of other proteins when arrested in PTEX but not in the PPM extractor. The lack of a co-block at the PPM may be due to the mechanism of how the PPM extractor receives proteins and its resulting architecture. As opposed to PTEX which obtains proteins from the PV, the TM substrates (arriving from a brefeldin A sensitive secretory pathway [18]) are already integral to the PPM and the extractor needs the ability to receive substrates laterally. This might be a reversible process, leading to disassociation of the substrate back into the membrane if its export is blocked and hence frees the extractor for other substrates. It should also be noted that blocking translocation generally requires a high saturation with arrested substrate [37]. A small proportion of free extractors may therefore already be sufficient to maintain export of other proteins.

The situation for the transport of exported TM proteins at the parasite periphery resembles that in certain plastids where the outer most membrane receives substrates in integral form from the Golgi after which they are translocated across the inner membranes, hence requiring extraction out of the first membrane [38,39]. How this extraction is achieved in these plastids is unclear [40] but the ERAD pathway in the ER membrane [41] and the Asi complex in the nuclear inner membrane [42,43] clearly demonstrate that a dislocation of membrane embedded proteins is possible. In absence of data on the nature of the PPM extractor, multiple configurations for the translocation set-up at the PPM and PVM can be envisaged (reviewed in [17]). In one possible scenario, the dislocation of TM proteins from the PPM is aided by PTEX, which is especially plausible for the substrates that are handed over to PTEX at the PVM while portions of the molecule are still being extracted out of the PPM. This is also supported by the capacity of HSP101 to disassociate from PTEX [10].

Many exported proteins, including the major virulence factor PfEMP1, contain TMs, and the identification of the PPM extractor will be crucial to understand how the export of these proteins is achieved. The translocation machineries in the malaria parasite PPM and PVM may have similar capacities to those in other systems such as the mitochondrial membranes that are remarkably versatile in regards to the types of substrates accepted and the destiny of delivery. Stable translocation intermediates will be essential to further unravel these mechanisms in malaria parasites.

## Materials and Methods

### Ethics statement

Animal handling and immunizations by Eurogentec were done in accordance with good animal practices according to the Belgian national animal welfare regulations for Eurogentec SA, Seraing and was under approval (CE/Sante/E/001) of the ethics committee of the Centre d’Economie Rurale (CER Groupe, Marloie, Belgium). Eurogentec follows the European Union directive 2010/63/EU (Welfare Legislation for laboratory animals).

### Plasmid constructs

To obtain mDHFR-GFP fusions expressed under the *crt*-promoter, inserts were PCR amplified with Phusion Polymerase (NEB) from 3D7 genomic DNA or cDNA using the primers listed in S1 Table and cloned as detailed in S2 Table. Inserts were digested with XhoI/AvrII and cloned into vector pARL2-DG [21] containing the blasticidine deaminase gene as resistance marker. To obtain SBP1-mDHFR-GFP-PHmut, the last 88 bp of GFP fused to a mutated PH domain [26] was amplified from a pARL1 plasmid containing this domain and cloned into SBP1-mDHFR-GFP using an internal GFP BstBI restriction site and XmaI. To swap the order of GFP and mDHFR in REX2mDHFR-GFP [9], REX2-GFP was amplified from REX2-GFP [35] with an additional primer-inserted NheI site after the GFP and cloned into pARL2 using XhoI and XmaI. mDHFR was inserted using NheI and XmaI to obtain REX2-GFP-mDHFR. MSRP-6 and REX-3 mCherry constructs were produced in vector pARL1-REX2-mCherry [31] containing human dihydrofolate reductase as resistance marker.

Wild type BPTI [20] (Uniprot accession P00974) and mutated BPTI [24] genes were commercially synthesised (GenScript) and inserted to replace mDHFR in vector pARL2 containing REX2-mDHFR-GFP using AvrII/KpnI, resulting in pARL2-REX2-BPTI-GFP. REX2+3C, SBP1, SBP1∆N, SBP1∆C, MAHRP1, and PTP1and REX3 were inserted into this vector using XhoI/AvrII to obtain the corresponding BPTI fusions. The SBP1∆N deletion insert was generated by overlap PCR using the primers listed in S1 Table. The REX2+3C insert was synthesized (GenScript), fusing three additional codon changed REX2-C termini (each encoding amino acids 61–94) to the construct. An additional SpeI restriction site was inserted before the 3C- termini. PTP1 was PCR amplified and cloned into pARL2 containing REX2-3C-BPTI-GFP and REX2-3C-mDHFR-GFP with Xho and SpeI to generate PTP1-3C-BPTI-GFP and PTP1-3C-mDHFR-GFP.

To obtain the skip peptide (T2A) vectors, the T2A sequence was inserted between GFP and mCherry in pARL2 GFP-mCherry flanked by XhoI and AvrII sites. MSRP6, KAHRP, REX-3 and STEVOR inserts were PCR amplified and cloned after T2A using AvRII/KpnI. SBP1mDHFR-GFP was amplified from the corresponding pARL2 construct and cloned before the skip peptide using XhoI/SpeI.

For HA tagging of the *exp*2 locus by single cross-over recombination the last 1000 bp of the *exp*2 gene were PCR amplified from 3D7 genomic DNA, adding a sequence encoding a 3xHA tag and an additional KpnI restriction site before the 3xHA tag with the reverse primer and cloned into vector pSLI-PfEHD2xFKBP (Genbank accession KU998257) using NotI/SalI to replace PfEHD2xFKBP, leading to an integration plasmid carrying N-terminally truncated *exp*2 without promoter. For HA tagging of the *hsp101* locus, the last 1000 pb of this gene were cloned NotI/KpnI to replace the *exp*2 fragment inserted into pSLI-PfEHD2xFKBP. To obtain a plasmid to tag the endogenous locus of *sbp1* with *mdhfr* and *gfp* base pairs 64–1181 of *sbp1* were PCR amplified and cloned NotI/AvrII into p-REX2mDHFR-int (Genbank accession KU998258), leading to an integration plasmid carrying a promoterless N-terminally truncated *sbp*1 gene fused with the sequence coding for mDHFR-GFP.

For GST fusion expression constructs, inserts were amplified with the primers listed in S1 Table and cloned with BamHI and XhoI into pGEX-6-P2 (GE healthcare).

Inserts of all plasmids were sequenced to confirm absence of undesired mutations.

### Parasite culture and transfection

*P*. *falciparum* parasites (3D7) were cultured in human 0+ erythrocytes (transfusion blood, Universitätsklinikum Hamburg-Eppendorf) with RPMI 1640 medium containing 0.5% AlbuMAX (Invitrogen) according to standard procedures [44]. Transfection of synchronized ring stages was performed with 100 μg of purified plasmid DNA (Qiagen) as described [45]. Positive selection was done with 4 nM WR99210 (Jacobus Pharmaceuticals) or 2 μg/ml Blasticidin S (Invitrogen). Double transfectant cell lines were produced by transfection of mDHFR-GFP constructs into WR resistant cell lines expressing pARL1-mCherry constructs and selected using Blasticidin S. Once a week these transfected cultures were treated with 4 nM WR to avoid loss of plasmid expressing the mCherry construct.

### Export arrest assays by ligand (WR) induced prevention of unfolding in mDHFR fusion expressing parasites

Parasite cell lines expressing mDHFR fusion proteins were synchronized with 5% sorbitol [46] to obtain ring stages before they expressed the transgene. Thereafter the parasites were grown for 24 hours in presence or absence (control) of 4 nM WR during which transgene expression occurred. The cells were either directly imaged, processed for immune fluorescence assays (IFA), lysed for parasite extracts, or processed for protease protection assays.

### Parasite growth assays

Percoll purified [47] late stage SBP1-mDHFR-GFP^endo^ parasites or sorbitol synchronized ring stages of the double transfected cell lines REX-2GFP-mDHFR/REX2-mCherry and REX-2-mDHFR-GFP/REX2-mCherry were washed with RPMI medium and brought back into culture with or without 4 nM WR. Giemsa-stained thin blood smears were collected every day and parasitemia and parasite stages were recorded. Data are representative of three independent experiments.

### Immunofluorescence assays (IFA)

IFAs were performed as described [48]. Briefly, parasites were washed twice with PBS and dried as a thin film on 10-well slides. Cells were fixed in 100% acetone for 30 minutes at room temperature. Antibodies were diluted in PBS/3%BSA and incubated for 1 hour, followed by 5 washes in PBS. Secondary antibodies were applied for 1 hour in PBS/3%BSA containing 1 μg/ml DAPI followed by 5 washes with PBS. Mounting medium (Dako) was added and the slide sealed with a coverslip for imaging. Dilutions of primary antibodies were: mouse anti-GFP 1:500 (Roche), rabbit anti-GFP (Thermo) 1:500, rabbit anti-KAHRP 1:500 (a kind gift of Prof. Brian Cooke), mouse anti-MSRP6 1:250 [7], rabbit anti-myc 1:500 (Cell Signaling Technologies), mouse anti-REX2 1:250 [45], rabbit anti MAHRP2 1:250 [[49], a kind gift of Prof. Hans-Peter Beck], rabbit anti-REX1 1:2000 (newly raised) and rat anti HA 1:500 (Roche). Secondary antibodies used were donkey anti-rabbit conjugated with Alexa Fluor-488, -594 or goat anti-rabbit conjugated with Alexa-647 and goat anti-mouse conjugated with Alexa Fluor-488, -594 or donkey anti mouse conjugated with Alexa-647 (Invitrogen) diluted 1:2000.

### Imaging

IFAs were directly imaged. For live cell imaging, the nuclei of GFP and mCherry-expressing parasites were stained with 1 μg/ml DAPI (Roche) for 5 min at 37°C and infected erythrocytes were imaged in medium as described [50]. Microscopy was done with a Zeiss Axio Scope M1 microscope equipped with a 100x/1,4 numerical aperture oil immersion lens. Images were collected with a Hamamatsu Orca C4742-95 camera and Zeiss AxioVision software. Images were processed in Corel PHOTO-PAINT X6.

### Newly raised antisera

Fragments of SERA-5 [aa 68–184 of PF3D7_0207600 [51]], Aldolase [aa 9–96 of PF3D7_1444800, [52]], REX1 [aa 332–596 of PF3D7_0935900, [53]], REX3 [aa 48–326 of PF3D7_0936300, [45]]; SBP1N [aa 13–208 of PF3D7_0501300, [54]] were expressed as GST fusion proteins, purified with GST-sepharose (GenScript) and antisera were commercially raised by Eurogentec. Single bands of the expected sizes were observed with the antisera in parasite extracts on Western blots (S8 Fig).

### Total parasite extracts

For total parasites extracts, parasites were released from RBCs using 0.03% saponin (Sigma) in PBS and washed twice with PBS. Proteins were then extracted with 4% SDS/0.5% Triton X-114/0.5 x PBS in presence of protease inhibitors (Roche). After centrifugation at 16’000g for 5 min, reducing SDS sample buffer was added to the supernatant which was then separated by SDS-PAGE.

### Protease protection assay

Protease protection assays were performed as described [9]. Percoll purified infected RBCs from 10 ml culture (5–10% parasitemia) were washed with RPMI medium and treated with 1 HU tetanolysin (Sigma) in 100 μl of Dulbecco PBS (DPBS) (Pan Biotech) at 37°C for 10 min. The permeabilised parasites were washed with DPBS, equally divided into three tubes that were incubated for 30 min on ice with either 100 μl DPBS alone (control), 100 μl DPBS containing 8 U/ml proteinase K (NEB), or 100 μl DPBS containing 0.03% saponin and 8 U/ml proteinase K, respectively. Reactions were quenched and proteins precipitated by adding trichloroacetic acid to 10% final concentration. The sample containing the precipitated proteins was centrifuged at 16'000 g for 20 minutes, washed twice with 100% acetone and resuspended in 50 μl 1x TE buffer and frozen at—20°C. Samples were thawed, SDS sample buffer was added and equal amounts were subjected to Western analysis.

### Western analysis

Protein samples were resolved by SDS-PAGE and transferred to nitrocellulose membranes (Protran) in a tankblot device (Bio-Rad) using transfer buffer (0.192 M Glycine, 0.1% SDS, 25 mM Tris) with 20% methanol. Blocking of membranes and dilutions of antibodies were done in PBS containing 5% skim milk. Washing steps were done with PBS. Primary antibodies were applied in the following dilutions: mouse anti-GFP (Roche) 1:1000; rabbit anti-GFP (Thermo) 1:2000; rat anti-mCherry, 1:1000 (Chromotek); rabbit anti-SERA5, 1:2000 (newly raised); rabbit anti-REX3, 1:2000 (newly raised); mouse anti-SBP1N, 1:2500 (newly raised); rabbit anti-aldolase, 1:4000 (newly raised); mouse anti-HSP101, 1:1000 [7]; rabbit anti-DHFR (Abcam), 1:1000; rat anti-HA (Roche) 1:4000. Horseradish peroxidase-conjugated secondary antibodies used were goat anti-rat (Dianova) and goat anti-mouse (Dianova) and diluted 1:3000 as well as donkey anti-rabbit (Dianova) 1:2500 and applied after three washes. Immunoreactions were detected by enhanced chemiluminiscence (Bio Rad/ Thermo) and detected on CEA RP NEW x-ray films (Agfa). For quantification of Western blot signals, band intensities were measured with a Chemi Doc XRS imaging system (Bio-Rad) and densitometry analyses were done with Image Lab Software 5.2 (Bio-Rad). Data are representative of three independent experiments.

### Co-immunoprecipitation assays

The EXP2-3xHA^endo^ cell line expressing SBP1-mDHFR-GFP, REX2-mDHFR-GFP or REX2-GFP-mDHFR, respectively, was sorbitol synchronized and ring stage parasites (~10% parasitemia) cultured with and without WR (4 nM) for 24 hours. The resulting trophozoites were harvested and washed twice with DPBS. For some experiments, cultures were crosslinked with 0.5 mM dithiobis (succinimidylpropionate) (DSP, 20 mM stock in DMSO) (Pierce) in DPBS for 30 minutes at room temperature and the reaction was quenched with PBS containing 25 mM Tris-HCl. Infected erythrocytes were purified in a Percoll gradient, washed with DPBS and lysed with RIPA buffer (10 mM Tris HCl pH 7.5, 150 mM NaCl, 0.1% SDS, 1% Triton) containing protease inhibitor cocktail (Roche) and 1 mM PMSF. After two freeze-thaw cycles, lysates were cleared by centrifugation at 16'000 g for 10 minutes. Supernatants were incubated with 25 μl of mouse monoclonal anti-HA beads (Pierce) or anti-GFP beads (Chromotek) for 3 hours at 4°C. Samples of input and post binding extracts were saved for SDS-PAGE. Beads were recovered by centrifugation and washed five times with RIPA buffer. Proteins were eluted in 50 μl 4 x SDS sample buffer at 85°C for 5 minutes. Equal volumes of input, post binding extract and bound fractions were subjected to Western analysis.

### Identification of EXP2 interacting proteins

Synchronised trophozoite cultures of EXP2-3XHA^endo^ and 3D7 parasites (100 ml each, 5% parasitemia), were harvested, washed twice with DPBS and dithiobis (succinimidylpropionate) (DSP, 20mM stock in DMSO) (Pierce) was added to 2 mM in DPBS for 30 minutes at room temperature. The reaction was quenched with 25 mM Tris-HCl pH 7.5 in DPBS for 10 minutes. Infected RBCs were purified in a Percoll gradient, washed with DPBS and lysed with RIPA buffer (10 mM Tris HCl pH 7.5, 150 mM NaCl, 0.1% SDS, 1% Triton) containing 2X protease cocktail inhibitors (Roche) and 1 mM PMSF. After two freeze-thaw cycles at -80°C, lysates were cleared by centrifugation at 16'000 g for 10 minutes. Supernatants were diluted 1:2 with RIPA buffer without detergents (10 mM Tris HCl pH 7.5, 150 mM NaCl) and equal volumes were incubated with 50 μl of anti-HA beads (Pierce) for 3 hours at 4°C with end-over-end rotation. Beads were recovered by centrifugation for 10 seconds at 11'000 rpm and washed five times with RIPA buffer. Cross linked interacting partners were released by ReCLIP [55] by incubating beads for 30 minutes at 37°C with RIPA buffer supplemented with 100 mM dithiotreitol followed by centrifugation to obtain the supernatant (ReCLIPed eluate, designated as Eluate 1). The beads were then incubated shortly with NaOH 50 mM, centrifuged and supernatant was saved (Eluate 2). Both eluates were then precipitated with trichloroacetic acid (TCA) 20% and analysed by mass spectrometry.

### Proteomic analyses

TCA Protein pellets were solubilized in lysis buffer (6 M urea, 2 M thiourea, 10 mM HEPES pH 8.0) by sonication for 10 min at 4°C. Proteins were reduced with 10 mM DTT for 10 min at room temperature and alkylated with 55 mM iodoacetamide for 20 min in the dark. Proteins were digested with 0.5 μg LysC (Wako) for 3h at room temperature. Samples were then diluted 1:4 with water and subsequently digested with mass-spectrometry grade trypsin (Promega) overnight at 32°C. Tryptic peptides were purified by SPE on a SepPAC-tC18 (Waters) according to the manufacturer's instructions, lyophilized and re-dissolved in 0.1% formic acid and spiked with 20 fmol/μL of yeast enolase 1 MassPREPTM protein digestion standard (Waters) prior to LC-MS analysis. Tryptic peptides were analysed using a nanoscale UPLC system (nanoAcquityUPLC) (Waters) coupled online to a Synapt G2-S HDMS mass spectrometer (Waters). Peptides were separated on a HSS-T3 1.7 μm, 75 μm x 250 mm reversed-phase column (Waters) using direct injection mode as described before [56]. Analysis was performed in positive mode ESI-MS using an ion-mobility enhanced data-dependent acquisition workflow (HD-DDA) described in detail previously [57]. The data were post-acquisition lock mass corrected using [Glu1]-Fibrinopeptide B. LC-MS data were processed using PEAKS v 7.5 (Bioinformatics Solutions Inc) searching against a combined database consisting of UniprotKB/Swissprot human database (UniProtKB release 2015_02) and UniProt Plasmodium 3D7 Reference Proteome, supplemented with common contaminant proteins, which was concatenated to a reversed decoy database, using the following search criteria for peptide identification: i) trypsin as digestion enzyme ii) up to three missed cleavages allowed iii) fixed carbamidomethylcysteine and variable methionine oxidation as modifications. Precursor and fragment ion mass tolerances were set to 15 ppm for precursors and 0.03 Da for fragment ions. The initial false discovery rate (FDR) for peptide identification was set to 1% in PEAKS based on a reversed decoy database search.

## Supporting Information

S1 Fig Irreversibility of export arrest of mDHFR fusion proteins and differing outcome of fusion with BPTI. (**A**) Representative IFA images of parasites expressing REX2-mDHFRmyc (constructs shown schematically above image panels) grown with (+WR) or without (control) WR and 5 h after removal of WR. A construct without GFP was chosen as this protein can after folding itself block translocation, which excludes this possibility as a reason for the irreversibility of the block. The PVM was co-stained using an antibody against ETRAMP5 (α-Etr5). The low amounts of signal detected at the Maurer’s clefts 5 h after removal of WR likely represents newly synthesized protein. (**B**) Schematic of the rational for using BPTI fusions of exported TM proteins to overcome the first translocation at the PPM and achieve arrest of export at the second translocation based on redox dependent folding of BPTI in the oxidizing environment of the PV. Features in the schematic are as in Fig 1E. (**C**) Representative live fluorescence images of the cell line expressing MAHRP1-BPTI-GFP (schematic of the construct shown above the panel). DIC, differential interference contrast. Size bars: 5 μm. (**D-E**) Model for the translocation of TM proteins between the PPM and PVM. Extraction out of the PPM is not hindered, as BPTI is unfolded in the reducing cytoplasm of the parasite (**D-E**, left). In proteins with a short C-terminus (**D**) fusion with BPTI results in a short distance between the TM and this domain. The TM then only reaches the PVM translocon once BPTI already emerged into the PV and its disulfide bridges can form in this oxidizing environment (middle panel). Further translocation across the PVM is then blocked (right). In contrast, in proteins with a long C-terminus (**E**), the distance between the TM and BPTI is long enough for the TM to reach the PVM translocon while BPTI is still unfolded in the parasite’s cytoplasm (**E**, middle). Concomitant extraction at the PPM and translocation at the PVM then leads to direct passage of the BPTI fusion protein into the host cell without exposing BPTI to the oxidizing environment of the PV and hence export is not inhibited (**E**, right). PPM extractor and PVM translocon may interact during this phase. The co-blocking activity of exported TM proteins fused to mDHFR likely depends on similar mechanistics related to the fact that these proteins can reach the PVM translocon during extraction while proteins with a short C-terminus remain in the PPM extractor only.
(TIF)

S2 Fig The export block of the REX2mCherry control depends on the expression of the mDHFR fusion protein and the co-blocked and the co-blocking constructs are found in the PV. (**A**) Representative live fluorescence images containing several infected RBCs of the cell line expressing SBP1-mDHFR-GFP together with the internal control REX2mCherry in the presence of WR (schematic of constructs is shown above the panels). The arrow shows a cell expressing only the mCherry construct but not SBP1-mDHFR-GFP (note that double transgenic cell lines frequently contain a proportion of parasites expressing only one of the transgenes). In contrast to the other cells that express SBP1-mDHFR-GFP, REX2mCherry is fully exported to the Maurer’s clefts. An image with reduced intensity (low) is shown to demonstrate the localization of the more intense cell at the bottom right. DIC, differential interference contrast. (**B**) Protease protection assay as explained in Fig 1E shows digestion of arrested (+WR) SBP1-mDHFR-GFP only if saponin to permeabilise the PVM is present. The size of the digested product is consistent with the protease resistant core (mDHFR-GFP), indicating no larger protected fragment and hence presence of the constructs in the PV. The same is the case for the co-blocked REX2mCherry (mCherry does not appear to form a stable core and was completely digested). SERA5 was used as a control for PVM integrity and REX3 as an indicator for efficient permeabilisation of the RBC membrane. The asterisk indicates the hemoglobin monomer (dimer and tetramer are also visible) which shows non-specific (antibody-independent) reaction with ECL often observed in the fraction containing host cell cytosol. Molecular weight standards are indicated (in kDa) on the left.
(TIF)

S3 Fig Comparability of skip peptide constructs with double transfectants. (**A**) Western blots demonstrate efficient skipping of the 2A containing constructs. Molecular weight standards are indicated (in kDa) on the left. Saponin supernatant (SN) and pellet (P) after Percoll enrichment are shown for the REX3mCherry expressing cell line. The calculated molecular weights are: SBP1-mDHFR-GFP: 87.5 kDa; STEVORmCherry: 60.85kDa; REX3mCherry: 64.5 kDa; MSRP6mCherry: 97.6 kDa; KAHRPmCherry: 98.2 kDa. As typical for many *P*. *falciparum* proteins, most products show a slower migration than expected. The asterisk indicates anti-GFP signal left over in an anti-mCherry reprobe of the same filter. Hashes indicate degradation products. (**B,C**) Representative live cell images of the double transgenic parasites expressing SBP1-mDHFR-GFP with either the PEXEL protein REX3mCherry (**B**) or the PNEP MSRP6 (**C**) from a second plasmid show comparable results to the same combinations expressed from a single mRNA using a skip peptide **(Fig 3A and 3C)**.
(TIF)

S4 Fig Additional co-blocking constructs and endogenous co-blocked proteins. (**A**) Representative live cell images on (+WR) and off WR (control) show that the export-blocked REX2-GFP-mDHFR induces a co-block of the co-expressed KAHRPmCherry in a double transgenic parasite line (schematic of constructs is shown above the panel). DIC, differential interference contrast. Size bar: 5 μm. A schematic of the co-block is shown to the right. Features of the schematics are as in Fig 2. (**B,C**) Representative images of an IFA with SBP1-mDHFR-GFP + REX2mCherry expressing parasites grown with (+WR) and without (control) WR, showing that SBP1mDHFR-GFP induces a WR-dependent co-block of endogenous exported proteins KAHRP and MSRP6 expressed late in the cycle (**B**) but not the proteins REX1 and MAHRP2 that are expressed and exported before the co-blocking transgene under the *crt* promoter is expressed (**C**). Size bars: 5 μm. Secondary antibodies for the signal shown in red were Alexa647 conjugated to avoid overlap with the left over mCherry signal of the REX2mCherry internal control. For (**B**) models for the co-block are shown to the right of the image. Features of the schematics are as in Fig 2.
(TIF)

S5 Fig The export of KAHRP-mDHFR is conditionally blocked if WR is added. Representative live cell images show that KAHRP fused to mDHFR-GFP is blocked in export in the presence of WR (+WR) and is exported in the absence of WR (control). The construct is shown schematically above the panels. The PEXEL motif is represented by a yellow box. SP, signal peptide. DIC, differential interference contrast. Size bars: 5 μm.
(TIF)

S6 Fig Analysis of SBP1-mDHFR-GFP^endo^. (**A**) PCR on genomic DNA of SBP1-mDHFR-GFP^endo^ and 3D7 (wt) parasites (as indicated) shows correct integration of the plasmid into the genome, leading to fusion of the endogenous *sbp-1* gene with *mdhfr* and *gfp*. A genome and a plasmid-specific primer were used each to confirm correct 5’ and 3’ integration. Primers (S1 Table) were SBP1-Int-check_F (3 bp after start ATG) with GFP42_rev to demonstrate 5’ integration (5’inte, 1815 bp) and SBP1-Int-check_R (23 bp after stop) with pARL55sense to demonstrate 3’ integration (3’inte, 1285 bp). Primers SBP1-Int-check_F and SBP1-Int-check_R were used to detect the unmodified original locus (1227 bp). (**B**) Western blot analysis detects SBP1-mDHFR-GFP but not unmodified SBP1 in SBP1-mDHFR-GFP^endo^ parasites while 3D7 contains only unmodified SBP1. Anti-mDHFR antibodies also detect the resistance marker (hDHFR) (arrow) expressed from the integrated plasmid in SBP1-mDHFR-GFP^endo^ parasites. Molecular weight standard is indicated in kDa. (**C**) Representative images of an IFA show that SBP1-mDHFR-GFP^endo^ co-blocks the endogenous early exported proteins REX2 and MAHRP2 in a WR-dependent manner. (**D**) Larger sections of the Giemsa stained smears shown in Fig 6.
(TIF)

S7 Fig Analysis of EXP1-3xHA^endo^. (**A**) PCR on genomic DNA of EXP2-3xHA^endo^ and 3D7 (wt) parasites (as indicated) shows correct integration of the plasmid into the genome, leading to fusion of the endogenous *exp-2* gene with a sequence coding for 3 HA tags. A genome and a plasmid-specific primer were used each to confirm correct 5’ and 3’ integration. Primers (S1 Table) were 5'EXP2fw (125 bp upstream of start ATG) with pARL_1_40rv to demonstrate 5’ integration (5’inte, 1729 bp) and 3’EXP2 rv (168 bp downstream of stop) with pARL55sense to demonstrate 3’ integration (3’inte, 1213 bp). Primers 5´EXP2fw and 3'EXP2rv were used to detect the unmodified original locus (1696 bp). (**B**) Western blot analysis using anti-HA antibodies detects triple HA tagged EXP2 in EXP-2-3xHA^endo^ but not in WT parasites. Expected molecular weight of EXP2-3xHA is 39,6 kDa. Molecular weight standard is indicated in kDa. (**C**) Western blot of immunoprecipitation samples carried out as shown in Fig 7C but using parasites that were first crosslinked with 0.5 mM dithiobis (succinimidylpropionate) (DSP). (**D**) Western blots of an IP experiment using HA binding beads with EXP-2-3xHA^endo^ parasites expressing the co-block inducing REX2-GFP-mDHFR. REX2-GFP-mDHFR co-purifies with EXP2-HA in parasites grown with WR (asterisk) but not in untreated controls. Input, total lysate before IP; post, lysate after IP. REX2-GFP-mDHFR was detected using anti-mDHFR antibodies as the sandwiched GFP is not well detected by the anti-GFP antibodies. (**E**) PCR on genomic DNA of HSP101-3xHA^endo^ and 3D7 (wt) parasites (as indicated) shows correct integration of the plasmid into the genome, leading to fusion of the endogenous *hsp101* gene with a sequence coding for 3 HA tags. A genome and a plasmid-specific primer were used each to confirm correct 5’ and 3’ integration. Primers (S1 Table) were 5'HSP101fw (473 bp upstream of start ATG) with pARL_1_40rv to demonstrate 5’ integration (5’inte, 4096 bp) and 3’HSP101rv (284 bp downstream of stop) with pARL55sense to demonstrate 3’ integration (3’inte, 1339 bp). Primers 5´HSP101fw and 3'HSP101rv were used to detect the unmodified original locus (4175 bp). (**F**) Western blot analysis using anti-HA antibodies detects triple HA tagged HSP101 in HSP101-3xHA^endo^ but not in WT parasites. Molecular weight standard is indicated in kDa. (**G**) Western blots of an IP experiment using HA binding beads with HSP101-3xHA^endo^ parasites expressing SBP1-mDHFR-GFP. SBP1-mDHFR-GFP is not coIPed in parasites grown with WR. Input, total lysate before IP; post, lysate after IP.
(TIF)

S8 Fig Western blots to show specificity of newly raised sera. Western blots of *P*. *falciparum* blood stage parasite protein extracts probed with the sera indicated beneath each blot. Molecular weight standard is indicated in kDa. Note that REX1 does not show a single band but one band between 100 and 130 kDa and several further bands with a lower molecular weight in a pattern that is typical for this protein[53].
(TIF)

S1 Table Primers used in this study. (PDF)

S2 Table Cloning strategy. (PDF)

S3 Table Extended MS Data of Fig 7C. (XLSX)
