# Supplementary figures and images for: Stable Translocation Intermediates Jam Global Protein Export in Plasmodium falciparum Parasites and Link the PTEX Component EXP2 with Translocation Activity
Source: PLoS Pathog. 2016 May 11;12(5):e1005618. doi: 10.1371/journal.ppat.1005618 (PMC4864081; doi:10.1371/journal.ppat.1005618)

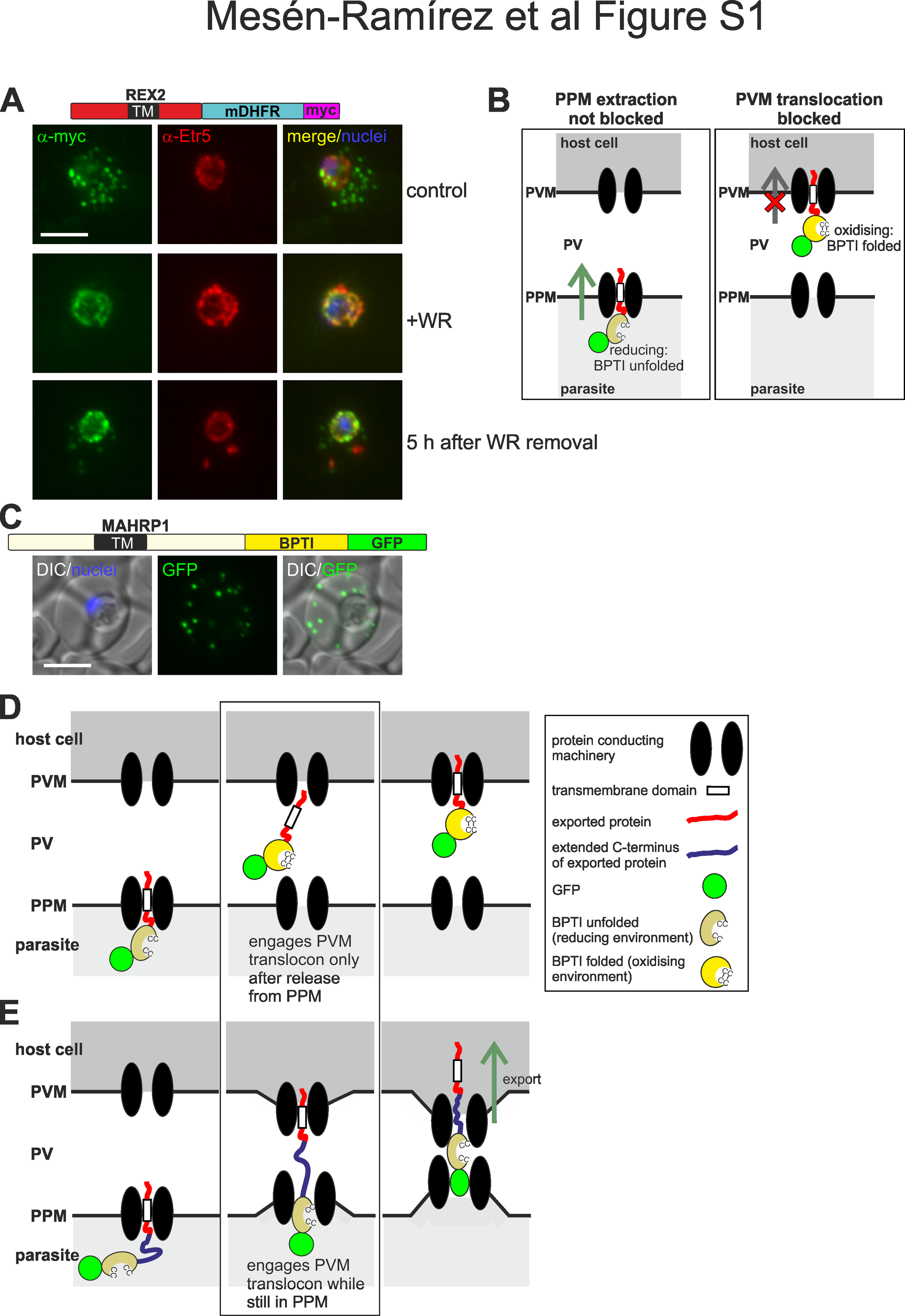

Supplement: S1 Fig — (A) Representative IFA images of parasites expressing REX2-mDHFRmyc (constructs shown schematically above image panels) grown with (+WR) or without (control) WR and 5 h after removal of WR. A construct without GFP was chosen as this protein can after folding itself block translocation, which excludes this possibility as a reason for the irreversibility of the block. The PVM was co-stained using an antibody against ETRAMP5 (α-Etr5). The low amounts of signal detected at the Maurer’s clefts 5 h after removal of WR likely represents newly synthesized protein. (B) Schematic of the rational for using BPTI fusions of exported TM proteins to overcome the first translocation at the PPM and achieve arrest of export at the second translocation based on redox dependent folding of BPTI in the oxidizing environment of the PV. Features in the schematic are as in Fig 1E. (C) Representative live fluorescence images of the cell line expressing MAHRP1-BPTI-GFP (schematic of the construct shown above the panel). DIC, differential interference contrast. Size bars: 5 μm. (D-E) Model for the translocation of TM proteins between the PPM and PVM. Extraction out of the PPM is not hindered, as BPTI is unfolded in the reducing cytoplasm of the parasite (D-E, left). In proteins with a short C-terminus (D) fusion with BPTI results in a short distance between the TM and this domain. The TM then only reaches the PVM translocon once BPTI already emerged into the PV and its disulfide bridges can form in this oxidizing environment (middle panel). Further translocation across the PVM is then blocked (right). In contrast, in proteins with a long C-terminus (E), the distance between the TM and BPTI is long enough for the TM to reach the PVM translocon while BPTI is still unfolded in the parasite’s cytoplasm (E, middle). Concomitant extraction at the PPM and translocation at the PVM then leads to direct passage of the BPTI fusion protein into the host cell without exposing BPTI to the oxidizing environm [file ppat.1005618.s001.tif]

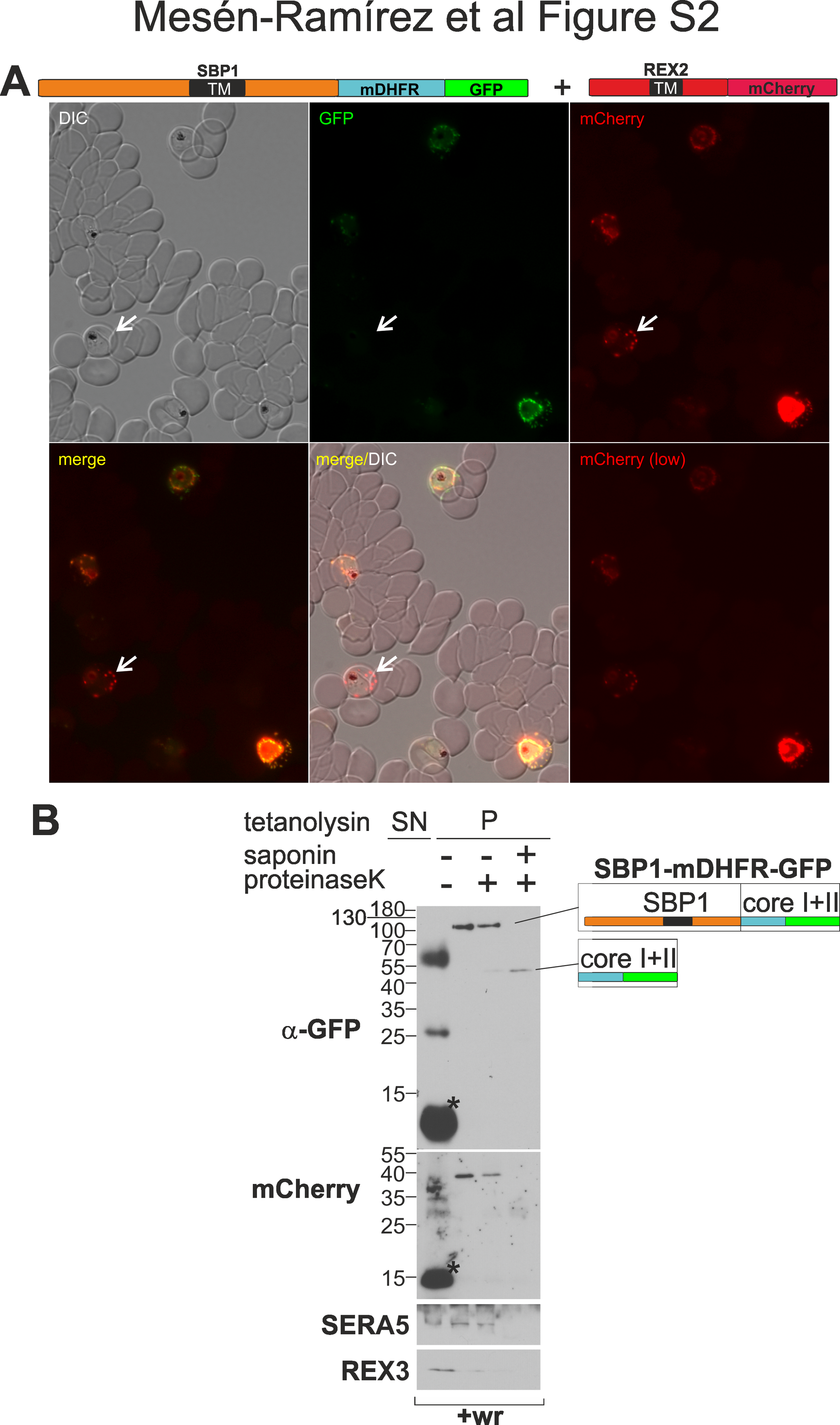

Supplement: S2 Fig — (A) Representative live fluorescence images containing several infected RBCs of the cell line expressing SBP1-mDHFR-GFP together with the internal control REX2mCherry in the presence of WR (schematic of constructs is shown above the panels). The arrow shows a cell expressing only the mCherry construct but not SBP1-mDHFR-GFP (note that double transgenic cell lines frequently contain a proportion of parasites expressing only one of the transgenes). In contrast to the other cells that express SBP1-mDHFR-GFP, REX2mCherry is fully exported to the Maurer’s clefts. An image with reduced intensity (low) is shown to demonstrate the localization of the more intense cell at the bottom right. DIC, differential interference contrast. (B) Protease protection assay as explained in Fig 1E shows digestion of arrested (+WR) SBP1-mDHFR-GFP only if saponin to permeabilise the PVM is present. The size of the digested product is consistent with the protease resistant core (mDHFR-GFP), indicating no larger protected fragment and hence presence of the constructs in the PV. The same is the case for the co-blocked REX2mCherry (mCherry does not appear to form a stable core and was completely digested). SERA5 was used as a control for PVM integrity and REX3 as an indicator for efficient permeabilisation of the RBC membrane. The asterisk indicates the hemoglobin monomer (dimer and tetramer are also visible) which shows non-specific (antibody-independent) reaction with ECL often observed in the fraction containing host cell cytosol. Molecular weight standards are indicated (in kDa) on the left. (TIF) [file ppat.1005618.s002.tif]

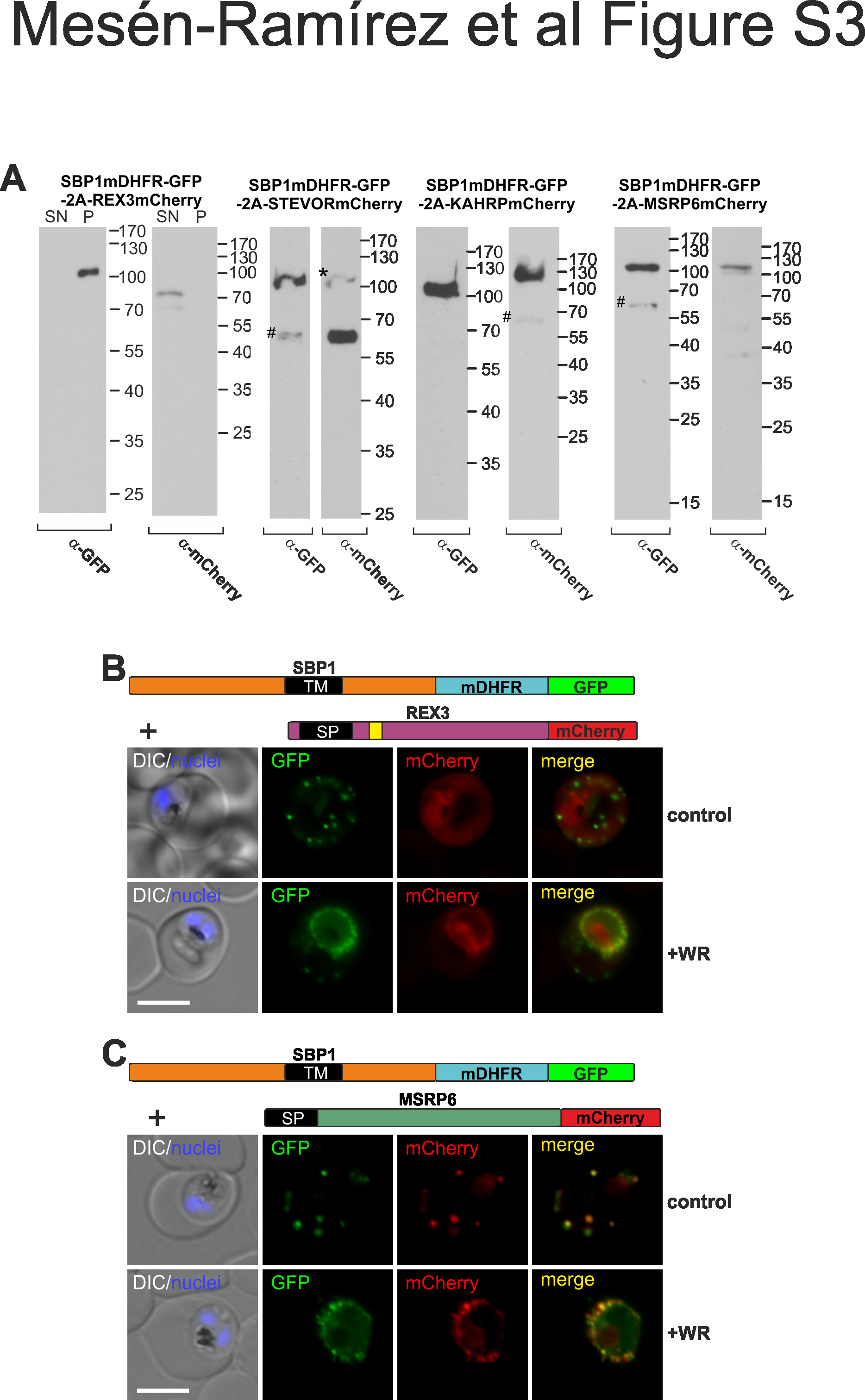

Supplement: S3 Fig — (A) Western blots demonstrate efficient skipping of the 2A containing constructs. Molecular weight standards are indicated (in kDa) on the left. Saponin supernatant (SN) and pellet (P) after Percoll enrichment are shown for the REX3mCherry expressing cell line. The calculated molecular weights are: SBP1-mDHFR-GFP: 87.5 kDa; STEVORmCherry: 60.85kDa; REX3mCherry: 64.5 kDa; MSRP6mCherry: 97.6 kDa; KAHRPmCherry: 98.2 kDa. As typical for many P. falciparum proteins, most products show a slower migration than expected. The asterisk indicates anti-GFP signal left over in an anti-mCherry reprobe of the same filter. Hashes indicate degradation products. (B,C) Representative live cell images of the double transgenic parasites expressing SBP1-mDHFR-GFP with either the PEXEL protein REX3mCherry (B) or the PNEP MSRP6 (C) from a second plasmid show comparable results to the same combinations expressed from a single mRNA using a skip peptide (Fig 3A and 3C). (TIF) [file ppat.1005618.s003.tif]

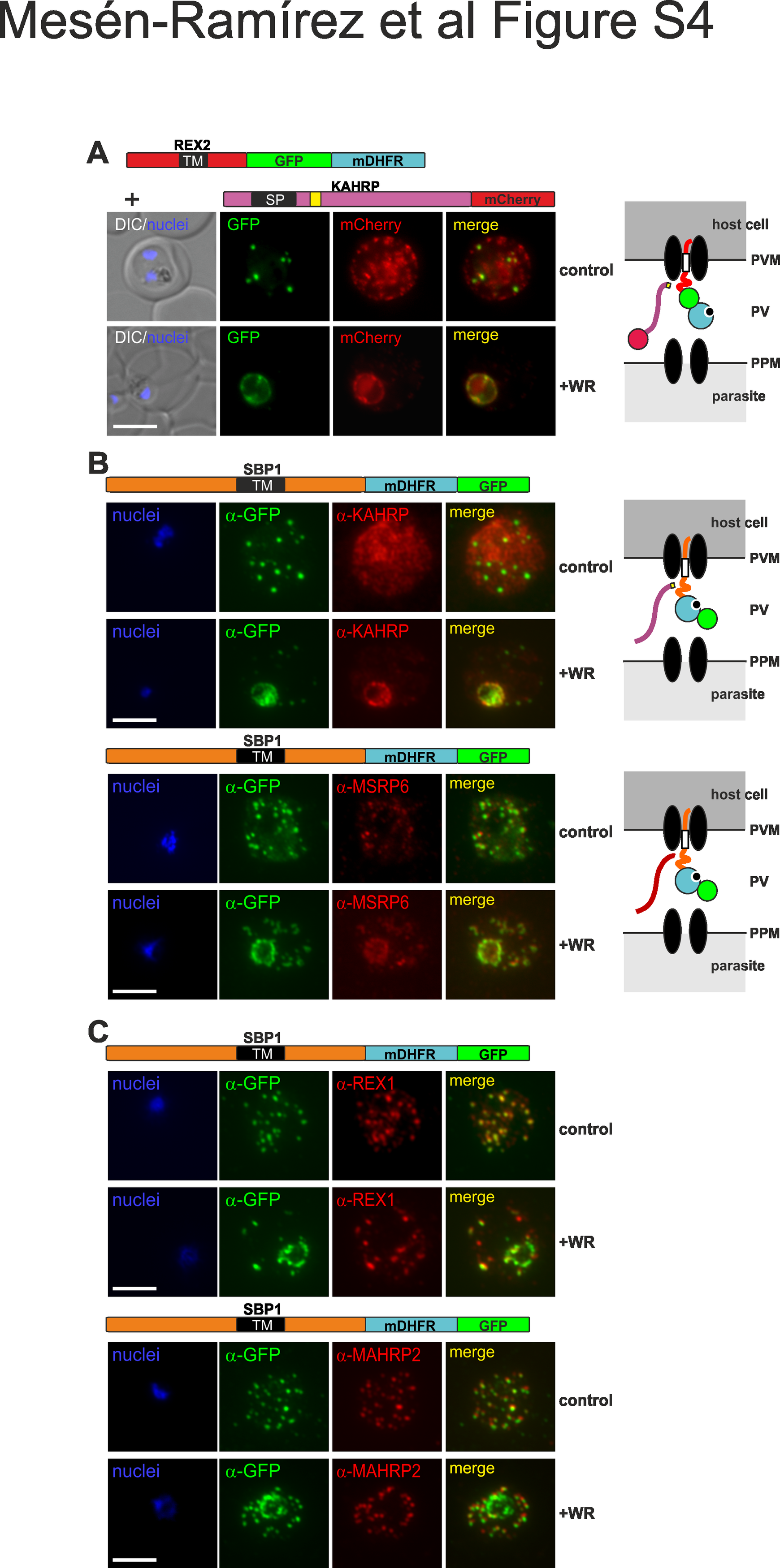

Supplement: S4 Fig — (A) Representative live cell images on (+WR) and off WR (control) show that the export-blocked REX2-GFP-mDHFR induces a co-block of the co-expressed KAHRPmCherry in a double transgenic parasite line (schematic of constructs is shown above the panel). DIC, differential interference contrast. Size bar: 5 μm. A schematic of the co-block is shown to the right. Features of the schematics are as in Fig 2. (B,C) Representative images of an IFA with SBP1-mDHFR-GFP + REX2mCherry expressing parasites grown with (+WR) and without (control) WR, showing that SBP1mDHFR-GFP induces a WR-dependent co-block of endogenous exported proteins KAHRP and MSRP6 expressed late in the cycle (B) but not the proteins REX1 and MAHRP2 that are expressed and exported before the co-blocking transgene under the crt promoter is expressed (C). Size bars: 5 μm. Secondary antibodies for the signal shown in red were Alexa647 conjugated to avoid overlap with the left over mCherry signal of the REX2mCherry internal control. For (B) models for the co-block are shown to the right of the image. Features of the schematics are as in Fig 2. (TIF) [file ppat.1005618.s004.tif]

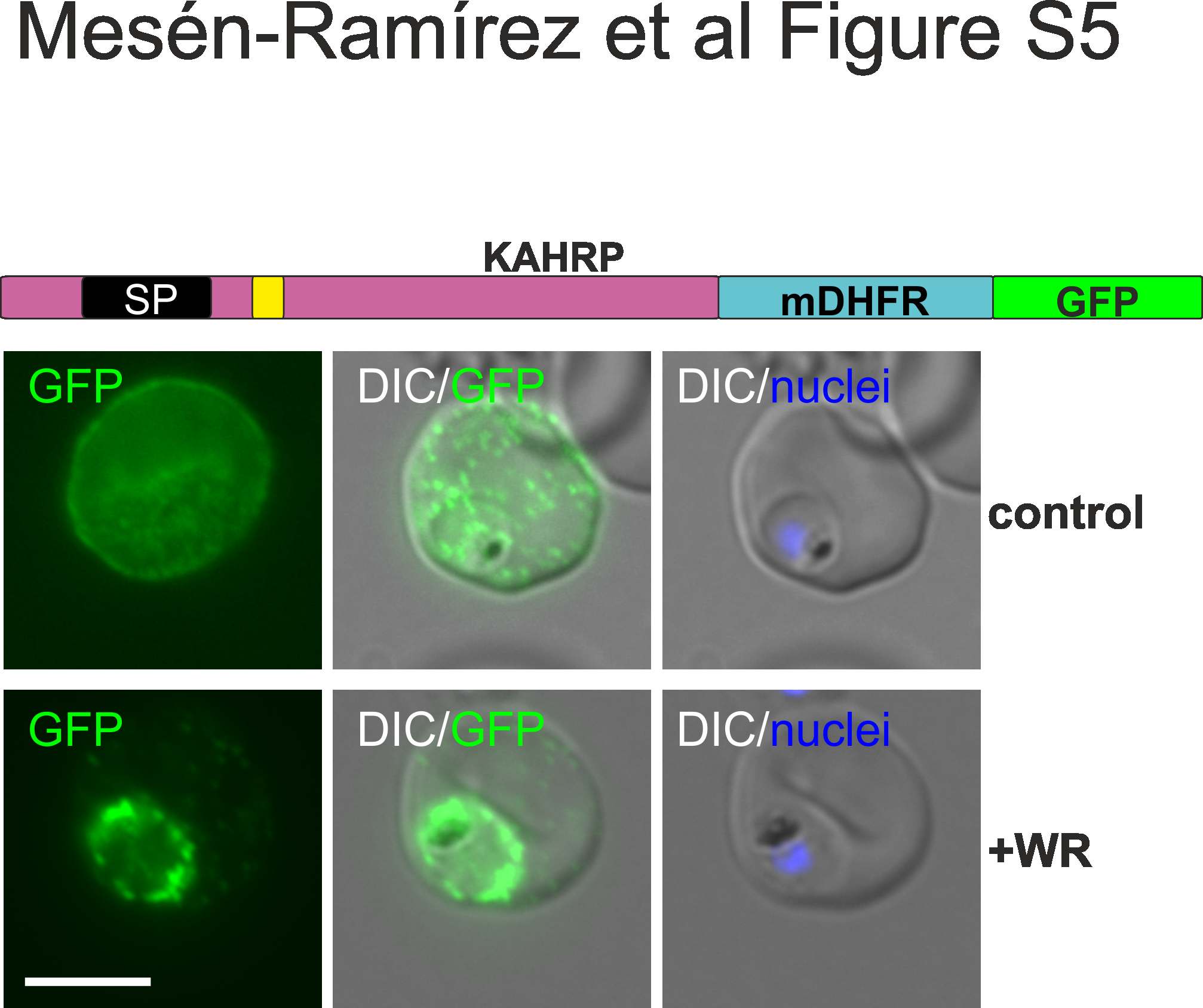

Supplement: S5 Fig — Representative live cell images show that KAHRP fused to mDHFR-GFP is blocked in export in the presence of WR (+WR) and is exported in the absence of WR (control). The construct is shown schematically above the panels. The PEXEL motif is represented by a yellow box. SP, signal peptide. DIC, differential interference contrast. Size bars: 5 μm. (TIF) [file ppat.1005618.s005.tif]

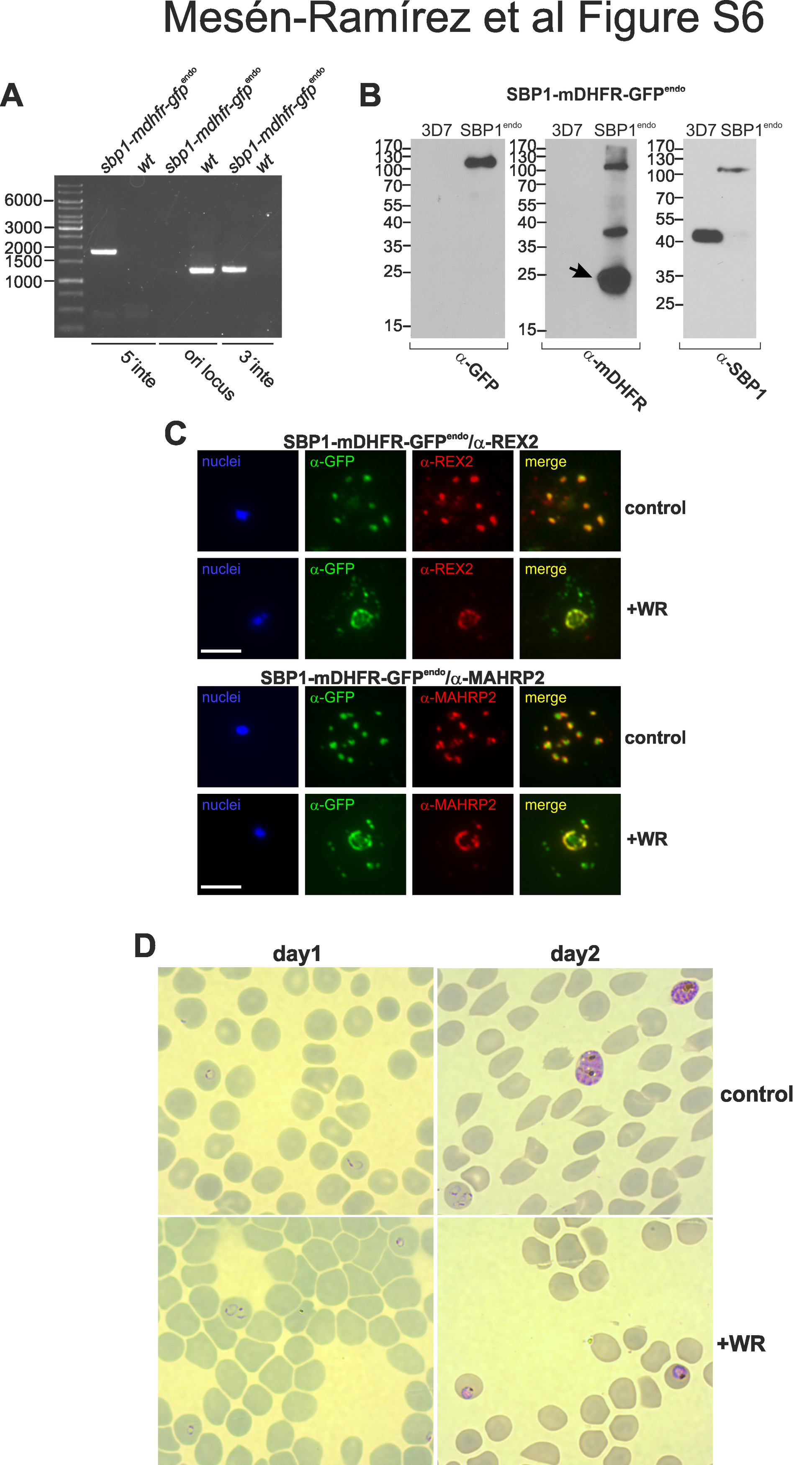

Supplement: S6 Fig — (A) PCR on genomic DNA of SBP1-mDHFR-GFPendo and 3D7 (wt) parasites (as indicated) shows correct integration of the plasmid into the genome, leading to fusion of the endogenous sbp-1 gene with mdhfr and gfp. A genome and a plasmid-specific primer were used each to confirm correct 5’ and 3’ integration. Primers (S1 Table) were SBP1-Int-check_F (3 bp after start ATG) with GFP42_rev to demonstrate 5’ integration (5’inte, 1815 bp) and SBP1-Int-check_R (23 bp after stop) with pARL55sense to demonstrate 3’ integration (3’inte, 1285 bp). Primers SBP1-Int-check_F and SBP1-Int-check_R were used to detect the unmodified original locus (1227 bp). (B) Western blot analysis detects SBP1-mDHFR-GFP but not unmodified SBP1 in SBP1-mDHFR-GFPendo parasites while 3D7 contains only unmodified SBP1. Anti-mDHFR antibodies also detect the resistance marker (hDHFR) (arrow) expressed from the integrated plasmid in SBP1-mDHFR-GFPendo parasites. Molecular weight standard is indicated in kDa. (C) Representative images of an IFA show that SBP1-mDHFR-GFPendo co-blocks the endogenous early exported proteins REX2 and MAHRP2 in a WR-dependent manner. (D) Larger sections of the Giemsa stained smears shown in Fig 6. (TIF) [file ppat.1005618.s006.tif]

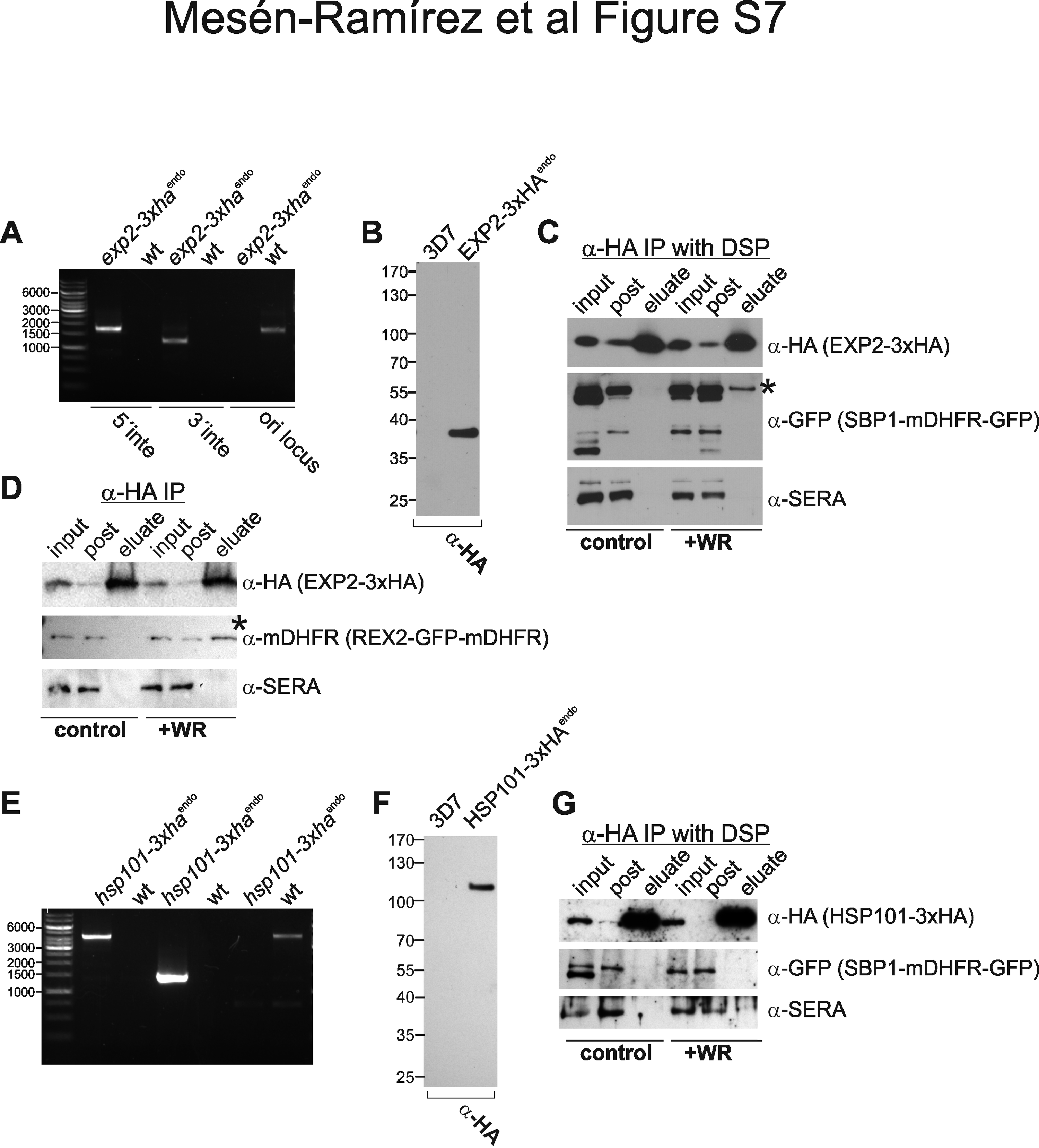

Supplement: S7 Fig — (A) PCR on genomic DNA of EXP2-3xHAendo and 3D7 (wt) parasites (as indicated) shows correct integration of the plasmid into the genome, leading to fusion of the endogenous exp-2 gene with a sequence coding for 3 HA tags. A genome and a plasmid-specific primer were used each to confirm correct 5’ and 3’ integration. Primers (S1 Table) were 5'EXP2fw (125 bp upstream of start ATG) with pARL_1_40rv to demonstrate 5’ integration (5’inte, 1729 bp) and 3’EXP2 rv (168 bp downstream of stop) with pARL55sense to demonstrate 3’ integration (3’inte, 1213 bp). Primers 5´EXP2fw and 3'EXP2rv were used to detect the unmodified original locus (1696 bp). (B) Western blot analysis using anti-HA antibodies detects triple HA tagged EXP2 in EXP-2-3xHAendo but not in WT parasites. Expected molecular weight of EXP2-3xHA is 39,6 kDa. Molecular weight standard is indicated in kDa. (C) Western blot of immunoprecipitation samples carried out as shown in Fig 7C but using parasites that were first crosslinked with 0.5 mM dithiobis (succinimidylpropionate) (DSP). (D) Western blots of an IP experiment using HA binding beads with EXP-2-3xHAendo parasites expressing the co-block inducing REX2-GFP-mDHFR. REX2-GFP-mDHFR co-purifies with EXP2-HA in parasites grown with WR (asterisk) but not in untreated controls. Input, total lysate before IP; post, lysate after IP. REX2-GFP-mDHFR was detected using anti-mDHFR antibodies as the sandwiched GFP is not well detected by the anti-GFP antibodies. (E) PCR on genomic DNA of HSP101-3xHAendo and 3D7 (wt) parasites (as indicated) shows correct integration of the plasmid into the genome, leading to fusion of the endogenous hsp101 gene with a sequence coding for 3 HA tags. A genome and a plasmid-specific primer were used each to confirm correct 5’ and 3’ integration. Primers (S1 Table) were 5'HSP101fw (473 bp upstream of start ATG) with pARL_1_40rv to demonstrate 5’ integration (5’inte, 4096 bp) and 3’HSP101rv (284 bp downstream of stop) with pARL55sense to demonst [file ppat.1005618.s007.tif]

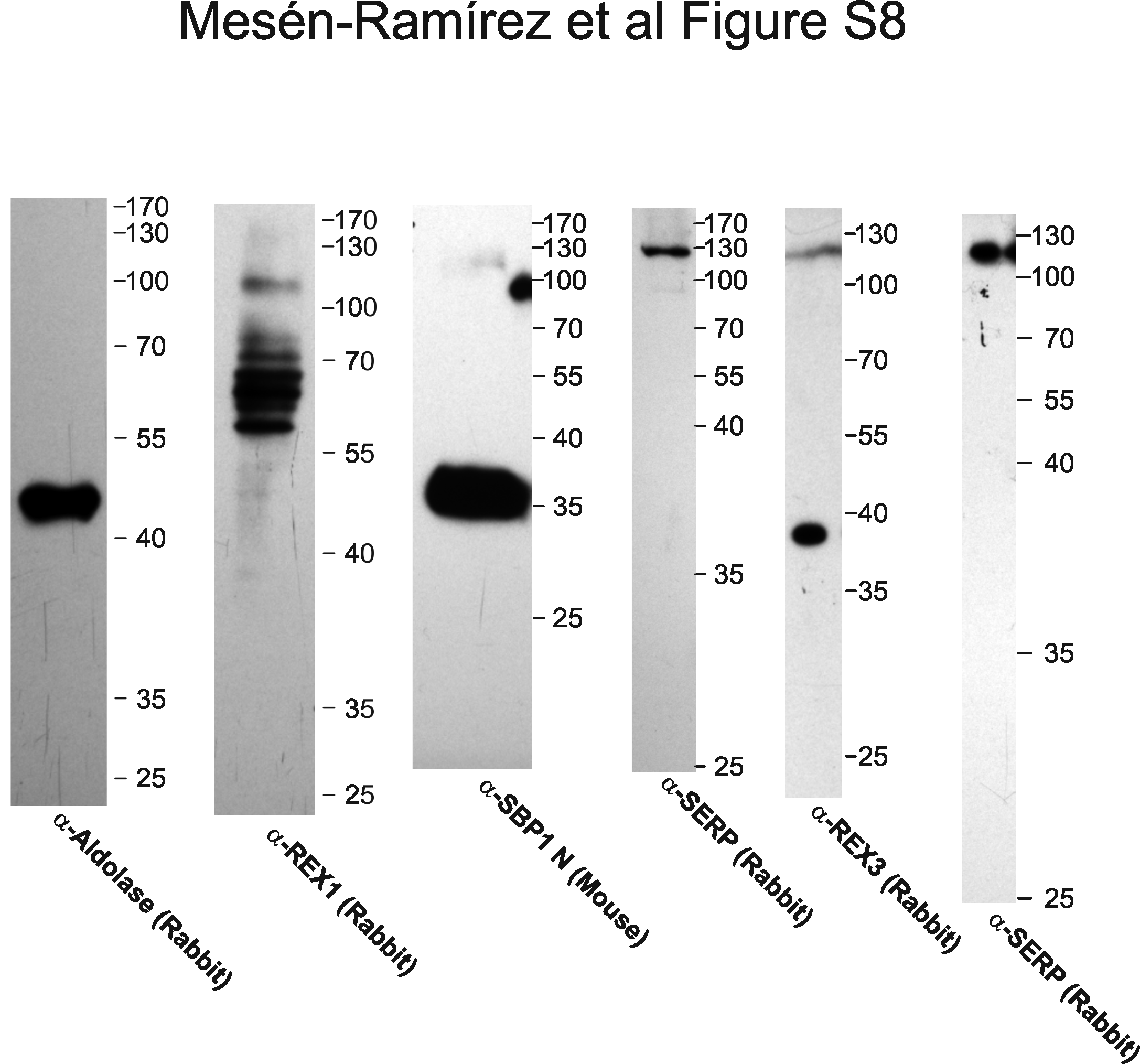

Supplement: S8 Fig — Western blots of P. falciparum blood stage parasite protein extracts probed with the sera indicated beneath each blot. Molecular weight standard is indicated in kDa. Note that REX1 does not show a single band but one band between 100 and 130 kDa and several further bands with a lower molecular weight in a pattern that is typical for this protein[53]. (TIF) [file ppat.1005618.s008.tif]
