# Supplementary material for: Stable Translocation Intermediates Jam Global Protein Export in Plasmodium falciparum Parasites and Link the PTEX Component EXP2 with Translocation Activity
Source: PLoS Pathog. 2016 May 11;12(5):e1005618. doi: 10.1371/journal.ppat.1005618 (PMC4864081; doi:10.1371/journal.ppat.1005618)
Supplement: S1 Table — (PDF) [file ppat.1005618.s009.pdf]

Table S1*. Primers used in this study

Primers	Sequence
SBP-1fw(XhoI)	GTCTctcgagATGTGTAGCGCAGCACGAGCATTTG
SBP-1rv(AvrII)	TCCTcctaggGGTTTCTCTAGCAACTGTTTTTGTTGTGG
SBP259AvRIIrv	TGATAGcctaggGTTTTGTTCTAGCATTTGTTC
SBP1 40rv	**GTCTCTGGTACTGCATCCTGTAATTG**TGTTGGTTCGTCGGCTAAATCAG
SBP1 211fw	**CAATTACAGGATGCAGTACCAGAGAC**AACCGAAAAATTGGCCGAAGTAGTTTCGGATGCAGCAATGTCCCCTATTCT TAGAGTACAATTTTTTGC
MAHRP1 Xho fw	GTCTctcgagATGGCAGAGCAAGCAGCAGTACAACCAGAAAG
MAHRP1rvAvRII	TCCTcctaggATTATCTTTTTTTTCTTGTTCTAATTTTGCTTTTTGGC
REX3 XhoI fw	CGGCctcgagATGCAAACCCGTAAATATAATAAGATGTTG
REX3 AvRII rv	TCCTcctaggTGAAGAACTTGTACTTGGTTTAGC
STEVOR XhoI fw	GTCTctcgagATGAAGATGTATAACCTTAAAATGTTATTG
STEVOR AvrII rv	CAGTcctaggCTTACATAAATGTTTCTTGCATTCATGTTTC
PTP1 XhoI fw	CTGTctcgagATGGTGAATAAAGATAATAGGAAAATTCATAAGGC
PTP1 rv AvrII	CAGTcctaggTTGGTTTTGTATATTTAAATTGTCATCTTGTTCC
PTP1 rv SpeI	CAGTactagtTTGGTTTTGTATATTTAAATTGTCATCTTGTTCC
KAHRP Xho fw	CTGTctcgagATGAAAAGTTTTAAGAACAAAAATACTTTGAGG
KAHRP AvRII rv	TCCTcctaggACCACAGCATCCTCTTTTCTTCTTTTTCTTTCC
GFP-PH mut fw BstBI	AGGAttcgaaAGATCCCAACGAAAAGAGAGACC
PH mut rv XmaI	CGAGcccgggTTACTTCAGGAAGTTCTGCAGCTCC
BPTI fwAvRII	GTCTcctaggTCAACACCAGGTTGTGATACATC
BPTI rvKpnI	TCCTggtaccTAAATTTTCCCATGGACCTATAGCAC
REX2 XhoI fw1	GTCTctcgagTTTATGAAAATGTATTTAGCTGAAATTTTTAGTTC
REX2rvAvrII Ext C-t	TCCTcctaggATGCTGCTGCTGATCGTACTGCTC
GFPrv NheI-XmaI	TCCTcccggggctagcTTTGTAGAGCTCATCCATGCCATGTG
mDHFR Spe-Xma rv	TCCTcccgggTTAactagtGTCTTTCTTCTCGTAGACTTCAAACTTATACTTGATGCC
mDHFR NheI fw	GTCTgctagcATGGTTCGACCATTGAACTGCATCG
pARL2 Xhofw	CTTCctcgagCAAAATGAAAAGTTTTATAACAAGAAATAAAACAGC
pARL2-2A-AvRIIrv	TCCTcctaggACTGATTGGTCCTGGATTTTCTTCTACATCTCCACATGTTAATAAACTTCCTCTTCCTTCTCCATAa ctagtCTTTGAGATTCGTCGG
GFPrvSpe	TCCTactagtTTTGTATAGTTCATCCATGCCATGTGTAATCCC
REX3 fw AvRII	CGGCcctaggATGCAAACCCGTAAATATAATAAGATGTTG
REX3 rv KpnI	TCCtggtaccTGAAGAACTTGTACTTGGTTTAGC
MSRP6 fw AvrII	AGGAcctaggATGAAAAGCAAAAAAATAATATGTTCATCTTGC
MSRP6 rv KpnI	TCCTggtaccTAATTTCGTGGGATTTAAAGC
KAHRP fw AvRII	CTTCcctaggATGAAAAGTTTTAAGAACAAAAATACTTTGAGG
KAHRP rv KpnI	TCCTggtaccACCACAGCATCCTCTTTTCTTCTTTTTCTTTCC
STEVOR fw1	**CTTTTTTGATTAATACTTTGG**CTTTGCCACATTATGATAATTATCAAAATAGCCATTATAATATAAACC
STEVOR fw2 AvRII	AGGAcctaggATGAAAATGTATAATTTGAAAATGTTGTTGTTTA**CTTTTTTGATTAATACTTTGG**
STEVOR rv Kpn	TCCTggtaccCTTGCATAAATGTTTCTTGCATTCATGTTTCC
REX3 fw KpnI	CGGCggtaccATGCAAACCCGTAAATATAATAAGATGTTG
REX3 rv AvrII	TCCTcctaggTGAAGAACTTGTACTTGGTTTAGC
MSRP6 fw KpnI	CAGCggtaccAAAAATGAAAAGCAAAAAAATAATATGTTCATCTTGC
MSRP6 rv AvRII	CAGCcctaggTAATTTCGTGGGATTTAAAGCTAAGTCC
STEVOR fw KpnI	GTCTggtaccATGAAGATGTATTACCTTAAAATGTTATTG
STEVOR rv AvrII	CAGTcctaggCTTACATAAATGTTTCTTGCATTCATGTTTCC
Rex1-332fwBamH1	CACGggatccATAGAACACTTAGAACACTTACAAGG
Rex1-596revXho1	TTGGctcgagATCTGATGTTGATGAACCTTTTGGAG
Aldolase-9-fwBamH1	CACGggatccAATGCCCCAAAAAAATTACCAGC
Aldolase-96-revXho1	TTGGctcgagACCGGCTTCATTCTTTTGAAATAATG
SBP1N-13fwBamH1	CACGggatccGATTTAGCCGACGAACCAACACAATTAC
SBP1N-208revXho1	TTGGctcgagGGCTTGTTTTGCAAAATCTGCAAAAAATTG
SERA5-68fwBamH1	CACGggatccCAAGGTAGTACGGGAGCAAGTCAACCC
SERA5-184revXho1	TTGGctcgagATTTGATGGTAGTTTTACATATTTTTTTTTTC
EXP2410 Not fw	CTCGgcggccgcTAATTTAACAATTAAAGATATTTATGAACACGG
EXP2HA rv1	**CGTACGGGTACATCGTAGCGTAATCTGG**AACATCGTATGGGTACATGGTggtaccTTCTTTATTTTCATCTTTTTTT TCATTTTTAAATAAATCTCC
3xHA-SalIrv2	TCCTgtcgacAGCATAATCTGGAACATCATATGGATACATAGTCGCGTAGTCCGGCACGT**CGTACGGGTACATCGTA GCGTAATCTGG**
5'EXP2fw	CTTTTATACCACCACTTCCCCTGTGTCATCG
3'EXP2rv	GATTGTTTCTCCATCAGATACTGTAC
HSP101NotIfw	CTCGgcggccgcTAATTTAAGAGATTCTGGTATGCCACTTGG
HSP101-HAKpnIrv	TCCTggtaccGGTCTTAGATAAGTTTATAACCAAGTTTTTAGC
5´HSP101fw	CCTTGTTTGTGATTTTAGTATGTAATTCGGAAC
3´HSP101rv	GGAACACACAAGTAACAATAAATTTACAAATGTG
pARL55sense	GGAATTGTGAGCGGATAACAATTTCACACAGG
pARL_1_40rv	CGAATAGCCTCTCCACCCAAG
NotI-SBP1_F	GTATgcggccgcTAGCAGGATGCAGTACCAGAGACAAC
AvrII-SBP1_R	CGCGcctaggACCTGAACCTGAACCGGTTTCTCTAGCAACTGTTTTTG
SBP1-Int-check_F	TGTAGCGCAGCTCGAGCATTTG
SBP1-Int-check_R	CAAATTCTCATTATTGTTGGCAC
GFP42_rev	CATCACCATCTAATTCAACAAG

* bases in small letters show restriction sites; bases in bold show overlapping regions for combination PCRs
