# Supplementary material for: Stable Translocation Intermediates Jam Global Protein Export in Plasmodium falciparum Parasites and Link the PTEX Component EXP2 with Translocation Activity
Source: PLoS Pathog. 2016 May 11;12(5):e1005618. doi: 10.1371/journal.ppat.1005618 (PMC4864081; doi:10.1371/journal.ppat.1005618)
Supplement: S2 Table — (PDF) [file ppat.1005618.s010.pdf]

Table S2. Cloning strategy

Construct	Primers	Template	Cloned into
SBP1-mDHFR-GFP	SBP-1fw(XhoI)<br>SBP-1rv(AvrII)	cDNA	pARL2-REX2mDHFR-GFP<br>(Grüring et. al 2012)
SBP1-mDHFR-GFP<br>PH mut	GFP-PH mut fw BstBI<br>PH mut rv XmaI	GFP PH mut<br>(Kruse et al, unpublished data)	pARL2<br>SBP-1mDHFR-GFP
REX2-GFP-mDHFR	REX2XhoI fw<br>GFPrv Nhe-XmaI<br>mDHFR Nhe fw<br>mDHFR Spe-XmaI	REX2-GFP<br>(Haase et al.,2009)<br>REX2mDHFR-GFP<br>(Grüring et. al 2012)	pARL2-REX2mDHFR-GFP<br>(Grüring et. al 2012)
REX2+3C-mDHFR-GFP	REX2XhoIfw<br>REX2rvAvrII Ext C-t	synthesized gene	pARL2-REX2mDHFR-GFP<br>(Grüring et. al 2012)
MAHRP1-mDHFR-GFP	MAHRP1 Xho fw<br>MAHRP1 AvRII rv	cDNA	pARL2-REX2mDHFR-GFP<br>(Grüring et. al 2012)
REX3-mDHFR-GFP	REX3 XhoI fw<br>REX3 AvRII rv	cDNA	pARL2-REX2mDHFR-GFP<br>(Grüring et. al 2012)
KAHRP-mDHFR-GFP	KAHRP Xho fw<br>KAHRP AvRII rv	cDNA	pARL2-REX2mDHFR-GFP<br>(Grüring et. al 2012)
PTP1-mDHFR-GFP	PTP1 XhoI fw<br>PTP1 rv AvrII	3D7 genomic DNA	pARL2-REX2mDHFR-GFP<br>(Grüring et. al 2012)
PTP1+3C-mDHFR-GFP	PTP1 XhoI fw<br>PTP1 rv SpeI	3D7 genomic DNA	pARL2-REX2 (3C) mDHFR-GFP
STEVOR-mDHFR-GFP	STEVOR_0900900 XhoI fw<br>STEVOR_0900900 AvrII rv	3D7 genomic DNA	pARL2-REX2mDHFR-GFP<br>(Grüring et. al 2012)
REX2-BPTIwt-GFP	BPTI fwAvRII<br>BPTI rv KpnI	synthesized gene	pARL2-REX2mDHFR-GFP<br>(Grüring et. al 2012)
REX2-BPTImut-GFP	BPTI fwAvRII<br>BPTI rv KpnI	synthesized gene	pARL2-REX2mDHFR-GFP<br>(Grüring et. al 2012)
SBP1-BPTIwt-GFP	SBP-1fw(XhoI)<br>SBP-1rv(AvrII)	cDNA	REX2-BPTIwt-GFP
MAHRP1-BPTIwt-GFP	MAHRP1 Xho fw<br>MAHRP1 rv AvrII	cDNA	REX2-BPTIwt-GFP
REX3-BPTI-GFP	REX3 XhoI fw<br>REX3 AvRII rv	cDNA	REX2-BPTIwt-GFP
ΔCSBP1-BPTIwt-GFP	SBP-1fw(XhoI)<br>SBP259AvRIIrv	cDNA	REX2-BPTIwt-GFP
ΔCSBP1-BPTImut-GFP	SBP-1fw(XhoI)<br>SBP259AvRIIrv	cDNA	REX2-BPTImut-GFP
ΔNSBP1-BPTIwt-GFP	SBP-1fw(XhoI) /SBP1 40rv (Product1a)<br>SBP1 211fw/ SBP-259rv(AvrII)(Product1b)<br>SBP-1fw(XhoI) /SBP259rv(AvrII)	cDNA<br>cDNA<br>Product1a + Product1b	REX2-BPTIwt-GFP
REX2+3C-BPTIwt-GFP	REX2XhoIfw<br>REX2rvAvrII Ext C-t	synthesized gene	REX2-BPTIwt-GFP
PTP1-BPTI-GFP	PTP1 XhoI fw<br>PTP1 rv AvrII	3D7 genomic DNA	REX2-BPTIwt-GFP
PTP1+3C-BPTI-GFP	PTP1 XhoI fw<br>PTP1 rv SpeI	3D7 genomic DNA	REX2-(3C) BPTIwt-GFP
MSRP6mCherry	MSRP6 fw KpnI<br>MSRP6 rv AvRII	MSRP6-GFP<br>(Heiber et al., 2013)	REX2mCherry<br>(Grüring et. al 2012)
REX3mCherry	REX3KpnIfw<br>REX3AvRIIrv	cDNA	REX2mCherry<br>(Grüring et. al 2012)
STEVORmCherry	STEVOR KpnI fw<br>STEVOR AvRII rv	3D7 genomic DNA	REX2mCherry<br>(Grüring et. al 2012)
pARL2-T2A-mCherry	pARL2 Xhofw<br>pARL2-2A-AvRIIrv	pARL1-GFP	pARL2 GFP-mCherry
SBP1-mDHFR-GFP-2A<br>REX3mCherry	SBP-1fw(XhoI)/GFPrvSpe<br>REX3 fw AvRII/ REX3 rv KpnI	SBP-1mDHFR-GFP<br>cDNA	pARL2 -T2A-mCherry
SBP1-mDHFR-GFP-2A<br>KAHRPmCherry	SBP-1fw(XhoI)/ GFPrvSpe<br>KAHRPfwAvRII/ KAHRPrvKpnI	SBP-1mDHFR-GFP<br>cDNA	pARL2 -T2A-mCherry
SBP1-mDHFR-GFP-2A<br>STEVORmCherry	SBP-1fw(XhoI)/ GFPrvSpe<br>STEVOR fw1/STEVOR fw2 AvRII<br>STEVOR rv Kpn	SBP-1mDHFR-GFP<br>3D7 genomic DNA	pARL2 -T2A-mCherry
SBP1-mDHFR-GFP-2A<br>MSRP6mCherry	SBP-1fw(XhoI)/ GFPrvSpe<br>MSRP6 fw AvRII/MSRP6 rv KpnI	SBP-1mDHFR-GFP<br>MSRP6-GFP	pARL2 -T2A-mCherry
REX2-GFP-mDHFR-2A<br>KAHRPmCherry	REX2XhoIfw/ mDHFR Spe-Xma rv<br>KAHRP fw AvRII/ KAHRP rv KpnI	REX2GFPmDHFR<br>cDNA	pARL2 -T2A-mCherry
GST-REX1	Rex1-332fwBamH1<br>Rex1-596revXho1	3D7 genomic DNA	pGEX-6-P2
GST-Aldolase	Aldolase-9-fwBamH1<br>Aldolase-96-revXho1	cDNA	pGEX-6-P2
GST-SBP1-N	SBP1N-131fwBamH1<br>SBP1N-208revXho1	SBP-1mDHFR-GFP	pGEX-6-P2
GST-SERA5	SERA5-68fwBamH1<br>SERA5-184revXho1	3D7 genomic DNA	pGEX-6-P2

EXP2-3xHA integration	EXP2 Not fw/ EXP2 HA rv1 (Product 1)<br>EXP2 Not fw/ 3xHA SalI rv2	3D7 genomic DNA<br>Product 1	pARL1-SLI
HSP101-3xHA integration	HSP101Not1 fw<br>HSP101Kpn1rv	3D7 genomic DNA	pARL1-SLI
SBP1-mDHFR-GFP integration	NotI-SBP1_F/AvrII-SBP1_R	3D7 genomic DNA	pARL2 mDHFR-GFP-hDHFR-BSD
